# Metagenomic inference of microbial community composition and function in the weathering crust aquifer of a temperate glacier

**DOI:** 10.3389/frmbi.2024.1488744

**Published:** 2024-11-13

**Authors:** Quincy Faber, Christina Davis, Brent Christner

**Affiliations:** Department of Microbiology and Cell Science, University of Florida, Gainesville, FL, United States

**Keywords:** metagenomics, ice algae, supraglacial, weathering crust aquifer, glacial ice

## Abstract

Bacterial, fungal, and algal communities that colonize aquatic systems on glacial ice surfaces mediate biogeochemical reactions that alter meltwater composition and affect meltwater production and storage. In this study, we sought to improve understanding of microbial communities inhabiting the shallow aquifer that forms seasonally within the ice surface of a glacier’s ablation zone (i.e., the weathering crust aquifer). Using a metagenomic approach, we compared gene contents of microbial assemblages in the weathering crust aquifer (WCA) of the Matanuska Glacier (Alaska, USA) to those recovered from supraglacial features and englacial ice. High abundances of Pseudomonadota, Cyanobacteriota, Actinomycetota, and Bacteroidota were observed across all samples, while taxa in class Gammaproteobacteria were found at significantly higher abundances in the weathering crust aquifer. The weathering crust aquifer samples also contained higher abundances of Dothideomycetes and Microbotryomyetes; fungal classes commonly observed in snow and other icy ecosystems. Phylogenetic analysis of 18S rRNA and *rbcL* gene sequences indicated high abundances of algae in the WCA that are closely related (> 98% and > 93% identity, respectively) to taxa of *Ancylonema* (Streptophyta) and *Ochromonas* (Ochrophyta) reported from glacial ice surfaces in Svalbard and Antarctic sea ice. Many functional gene categories (e.g., homeostasis, cellular regulation, and stress responses) were enriched in samples from the weathering crust aquifer compared to those from proximal englacial and supraglacial habitats, providing evidence for ecological specialization in the communities. The identification of phagotrophic phytoflagellate taxa and genes involved in mixotrophy implies that combined phototrophic and heterotrophic production may assist with persistence in the low light, low energy, and ephemeral conditions of the weathering crust environment. The compositional and functional differences we have documented indicate distinct microbial distributions and functional processes occur in the weathering crust aquifer environment, and we discuss how deciphering these nuances is essential for developing a more complete understanding of ecosystem biogeochemistry in supraglacial hydrological systems.

## Introduction

The seasonal generation of meltwater on the surface of glaciers and ice sheets creates liquid water that allows solute transport, microbial metabolism, and biogeochemical transformations in snow- and ice-based aquatic ecosystems during the melt season. Examples of these include algal- and cyanobacterial-based communities that colonize ice (Yallop et al., 2012; Remias et al., 2012), snow (Hoham and Duval, 2001), cryoconite holes (Hodson et al., 2010), and supraglacial streams (Irvine-Fynn et al., 2012; Foreman et al., 2013). In the ablation zone (i.e., where annual melting leads to net loss of ice from the glacier) on western portions of the Greenland Ice Sheet, the growth of darkly pigmented algae generates large blooms (Williamson et al., 2020) that accelerate ice melt (Yallop et al., 2012; Remias et al., 2012). Cryoconite holes are water-filled depressions of dark sediment that have melted into the ice surface and contain communities composed of phototrophs, heterotrophic bacteria, and fungi, as well as ciliates, tardigrades, and rotifers (Cook et al., 2016a; Zawierucha et al., 2015). Streams flowing in channels on the glacier surface are also known to be biogeochemical hotspots, with microbial metabolism altering nutrient compositions of water during its transport through the supraglacial hydrological system (Scott et al., 2010). Research on the microbiomes and biogeochemistry of glacial environments has accelerated over the last decade due to the realization of the rapid changes currently underway (Constable et al., 2023) and an increased awareness of their contributions to global carbon cycling (Anesio et al., 2017; Wadham et al., 2019).

Water observed in standing pools or flowing in streams on a glacier’s surface represents the portion of the meltwater that can be most rapidly exported to subglacial and proglacial environments (Varliero et al., 2023). This meltwater is sourced from water-bearing porous ice in the near-surface that forms a perched aquifer termed the weathering crust aquifer (WCA; Karlstrom et al., 2014; Cook et al., 2016b; Cooper et al., 2018). The WCA forms and persists in the ablation zone as seasonal increases in air temperature and incident shortwave radiation cause warming and internal melting to depths of 30 to 100 cm in the ice (Hoffman et al., 2014; Cooper et al., 2018; Irvine-Fynn et al., 2021). Given the presence of liquid water and light, photosynthesis is possible in the WCA (Hodson et al., 2013; Irvine-Fynn and Edwards, 2014; Christner et al., 2018). Flow through tortuous paths in the porous ice tends to increase residence time relative to water in other supraglacial environments (Stevens et al., 2018); conditions that may also enhance microbial interactions, biogeochemical processing, and production of new biomass. This contention is supported by data from western Greenland that estimate 37 kg km^−2^ of the cellular carbon produced in the WCA is exported to proglacial waters every melt season, representing a significant and overlooked contribution to global carbon cycling (Irvine-Fynn et al., 2021). The hydrological properties of the weathering crust decrease ice albedo (Tedstone et al., 2020) and enhance retention of nutrients that support algal growth (Holland et al., 2019); however, little is known about microbial nutrient processing in the WCA. Though previous studies have documented bacterial communities in WCA pore waters that differ from those associated with other supraglacial features (Christner et al., 2018; Rassner et al., 2024), it is not well understood if the WCA ecosystem is functionally distinct or contains similar metabolisms and taxa to those in proximal supraglacial microbial communities.

Given that environmental changes in glaciated regions have important social, scientific, and environmental impacts globally (e.g., Heckmann et al., 2016), there is a critical need to improve understanding on the microbiological sources and sinks of nutrients cycled in ice-based ecosystems and biogeochemical consequences of the meltwater discharged to proglacial environments. The ubiquity of algae and cyanobacteria in supraglacial communities (e.g., Anesio et al., 2009; Cook et al., 2012) has led to an assumption that autotrophic activity is driving carbon metabolism, yet studies of cryoconite holes have shown many to be net heterotrophic (Hodson et al., 2010). Algal blooms on glacier surfaces (Cook et al., 2012; Williamson et al., 2018) that coincide with higher abundances of presumably heterotrophic bacteria (Nicholes et al., 2019) and fungi (Perini et al., 2019) suggest these net autotrophic communities fuel supraglacial food webs. While certain fungi are known to be parasites of glacier algae (i.e., Chytridiomycota; Perini et al., 2022; Nakanishi et al., 2023), few studies have explored the diversity and role of fungal communities in supraglacial environments (Perini et al., 2022; Fiołka et al., 2021).

A previous study of the temperate Matanuska Glacier (Alaska, USA) during the melt season inferred internal melting from solar radiation to a maximum depth of ~2 m, estimated that ~10% of the surface ice volume was liquid water (200 L m^-2^), and revealed meltwaters containing growing microbial populations with estimated doubling times of ~two weeks (Christner et al., 2018). Sequencing and analysis of amplified small subunit (SSU) rRNA genes showed that samples from the near surface, water-bearing ice were enriched with certain taxa within the Alphaproteobacteria, Bacteroidota, and Cyanobacteriota, and contained a bacterial community composition distinct from assemblages detected in supraglacial waters and englacial ice. Phylotypes closely related to common ice and snow algae (i.e., *Ancylonema nordenskioeldii*, *Mesotaenium* sp. AG-2009-1, *Ochromonas* sp. CMP1899, and *Chrysophyceae* sp. 176) were also identified. However, the SSU rRNA gene data are poorly suited for resolving subtle ecological variation in natural populations and predicting the metabolic potential of the communities. In this study, we tested the hypothesis that the physical and hydrogeochemical conditions in the WCA select for a community comprised of bacterial and eukaryotic taxa distinct from the varieties in proximal supraglacial features. To do this, we used a metagenomic approach to more thoroughly examine community composition and functional gene content of supraglacial communities associated with the Matanuska Glacier. The purpose of our study was to holistically examine the bacterial, archaeal, and eukaryotic communities of the WCA, examine the complement of genes they possess, make predictions of their functional characteristics, and compare them to proximal supraglacial habitats. Indeed, the bacterial, algal, and fungal assemblages in the WCA possess distinct compositions and structures, and we discuss how many of the functions identified are highly relevant to survival in the low temperature, light, and nutrient conditions of an ecotone bridging the supraglacial and englacial environment.

## Materials and methods

### Site description and sample collection

The Matanuska Glacier is a temperate valley glacier in the Chugach Mountains of south-central Alaska, USA. The samples analyzed in this study were collected during June and July of 2014 and 2015 at a field site (61°42′9.3’’ N, 147°37′23.2’’ W) on the glacier’s western margin and ~8 km up glacier from the terminus. During the study, ten boreholes (ranging from 4 to 30 m in depth) were melted into the glacier that allowed meltwater primarily sourced from the englacial ice and the near-surface WCA to be sampled (Christner et al., 2018).

**Figure 1:**
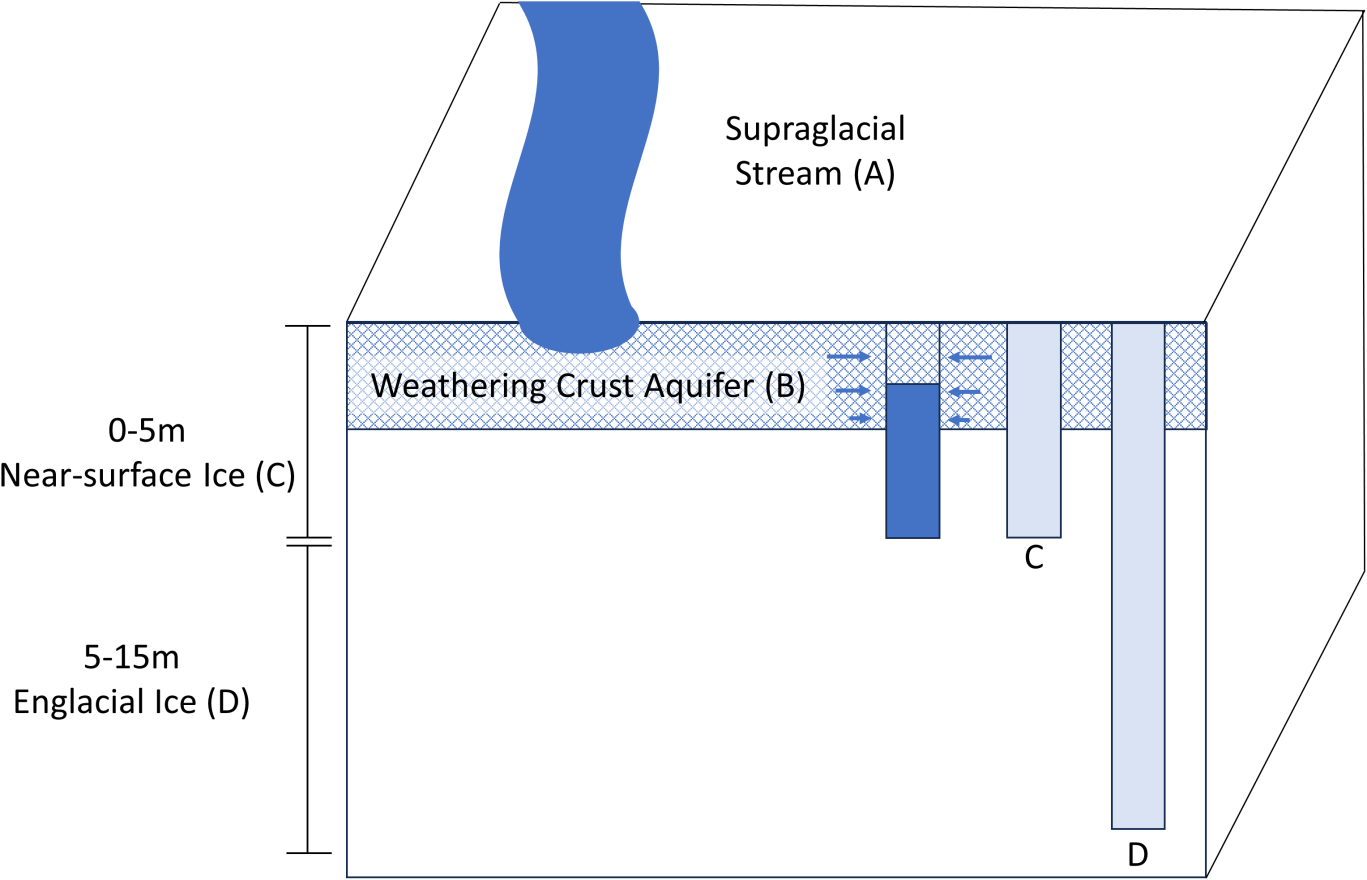
Schematic of the supraglacial and englacial features sampled in this study from the ablation zone of the Matanuska Glacier including the supraglacial stream **(A)**, weathering crust aquifer **(B)**, near-surface ice **(C)**, and englacial ice **(D)**.

The range of borehole depths examined were chosen to collectively access the transition of weathered ice on the surface into the underlying sequence of englacial ice. To sample englacial ice, we selected depths exceeding 5 m because it provided high confidence that the ice had not been affected by surface effects (i.e. solar radiation and melting). Subsequent modeling of ice temperature and downward radiation data implied that liquid water from internal melting was unlikely at depths > 2 m (Christner et al., 2018). Extensive detail on the methods used to generate the boreholes and sample the meltwater have been described previously (Christner et al., 2018). Briefly, the near-surface ice (depth < 5 m) and englacial ice (depths of 5 to 15 m; **Figure 1**) were sampled by melting boreholes into the surface using equipment that was precleaned with 3% (v/v) hydrogen peroxide. This generated meltwater that was subsequently transferred to the surface using clean tubing and a peristaltic pump (**Figures 1C, D**). Two of the boreholes (BH6 and BH7) were melted using technology that allowed sampling at discrete depths (1.1 to 15.4 m; **Supplementary Table S1**). To sample the WCA, the water generated from melting boreholes of ~4 m depth was discarded, and following refill with water that had percolated laterally through the near surface ice (**Figure 1B**), the material that collected in the borehole was filter concentrated and preserved. A supraglacial stream ~100 m upstream of the primary sampling site was also sampled (**Figure 1A**). For most samples, 20 to 60 L of meltwater was filtered using a peristaltic pump (**Supplementary Table S1**); however, several samples were obtained from large volumes of water (~400 L) with a deployable borehole filtration system (Christner et al., 2014). Cells in the water samples were concentrated on 90 mm or 142 mm Supor 0.2 µm pore size filters (Pall 66549), preserved in 40 mM EDTA pH 8.0, 50 mM Tris pH 8.3, and 0.73 M sucrose, shipped frozen overnight from Alaska to the laboratory, and were stored at -80°C until analyzed.

### DNA extraction and sequencing

DNA was extracted from cells concentrated on the filters using the PowerWater DNA Isolation Kit (MO BIO Laboratories, Inc.) and the modifications described by Christner et al. (2018). The extracted DNA was quantified with the dsDNA HS Assay Kit (ThermoFisher Scientific) and a Qubit 3 fluorometer. Samples with low genomic DNA yields (< 0.3 ng mL^-1^; indicated in **Supplementary Table S1**) were amplified using the GenomiPhi V3 DNA Amplification kit (Cytiva). Following the manufacturer’s protocol, 10 µL of the DNA sample was added to 10 µL of denaturing buffer, followed by addition to the Ready-To-Go GenomiPhi cake for amplification at 30°C using a Bio-Rad C1000 Touch thermal cycler. After 1.5 h, the reaction was terminated by heating to 65°C for 10 min and the samples were stored at 4°C for subsequent processing.

Library preparation and sequencing were performed at the ICBR Next Gen DNA Sequencing Core at the University of Florida. Due to low DNA extraction yields for many of the samples, libraries were constructed using the Ovation Ultralow system v2 kit. Quality and concentration of the libraries were checked using an Agilent 4150 TapeStation system and Qubit fluorometer, respectively. Paired-end sequencing was conducted on the NextSeq500 2x150 MID platform (Illumina, Inc.).

### Read processing and assembly

The sequencing reads were paired and low-quality reads were removed using Trimmomatic 0.39 (LEADING:3 TRAILING:3 SLIDINGWINDOW:4:15 MINLEN:36; Bolger et al., 2014). For analyses with unassembled sequences, paired-end reads were merged using BBmerge in BBMap Version 37.62 (Bushnell et al., 2017). *De novo* assembly of reads into contigs and scaffolds was performed using SPAdes 3.13.0 and default parameters (Bankevich et al., 2012). MetaBAT (V2.13; Kang et al., 2019) was used to create bins that were assessed for completeness using CheckM (V 1.1.2; Parks et al., 2015).

### Taxonomic and functional analysis

The reads containing sequence derived from SSU rRNA genes were extracted using Parallel Meta 3.6. OTUs were assigned in QIIME2 V2023.7 using the SILVA 138 99% OTUs classifier trained on full-length sequences and taxonomy (Bokulich et al., 2018; Quast et al., 2013). Only samples containing > 100 mapped rRNA gene sequences were used for alpha and beta diversity analysis (**Supplementary Table S3**). Simpson’s Diversity Index and Good’s estimator of coverage were calculated for each sample in R using the vegan package and the QsRutils package, respectively. Reads containing the ribulose-1,5-bisphosphate carboxylase/oxygenase large subunit gene (*rbcL*) and the internal transcribed spacer 2 region (ITS2) were identified using BlastN v2.9 (Camacho et al., 2009) with a custom database of all *rbcL* genes and fungal ITS2 sequences in the NCBI Reference Sequence database as of August 2020. QIIME 1.9.1 was used for reference-based OTU assignment and taxonomic identifications were conducted using the custom databases from RefSeq at the 97% sequence identity threshold (Caporaso et al., 2010). Nonmetric multidimensional scaling (NMDS) plots based on Bray-Curtis distance (iterations = 20) were visualized using the vegan package in R. Abundance tables based on the number of reads identified as SSU rRNA and *rbcL* genes, as well as the ITS2 region, were visualized using the ggplot2 package in R (v4.1.2; R Core Team 2021).

The sequences of 18S rRNA and *rbcL* genes were identified in the contigs using BlastN v2.9 (Camacho et al., 2009) and examined with the assistance of a database created using nucleotide sequences available in NCBI and with an E-value cutoff < 1e-100. To generate alignments of partial 18S rRNA genes for phylogenetic analysis, the sequences were aligned based on secondary structure using the SINA aligner (v1.2.12, Pruesse et al., 2012). For the *rbcL* sequences, the top five BLAST hits for each query sequence were aligned using MAFFT v7 (Kuraku et al., 2013). The alignments were used for phylogenetic analysis by maximum likelihood, using the Jukes-Cantor model with 100 bootstraps, and to generate distance matrices in MEGAX v11.0.10 (Kumar et al., 2018).

A kmer-based strategy was used to identify the contigs as prokaryotic, or eukaryotic with Eukrep (West et al., 2018). Functional predictions of bacterial and archaeal genes encoded by the contigs were conducted using Prodigal v2.6.3 (Hyatt et al., 2010) and eukaryotic genes were predicted using MetaEuk v6 (Levy Karin et al., 2020). Genes were aligned to the SWISS-PROT database using BlastX in DIAMOND v2.0.9.147 (Buchfink et al., 2021; Bairoch and Apweiler, 2000) and their taxonomy was assigned using MEGAN6 (Huson et al., 2016). All metagenomic reads were mapped to the predicted genes using Bowtie2 v2.4.5 (Langmead and Salzberg, 2012), duplicates were removed using SAMtools v1.18 (Li et al., 2009), and the “pileup.sh” script in BBMap v38.9 (Bushnell et al., 2017) was used to summarize the number of reads mapped to each gene. The outputs of these analyses were exported to R for statistical analysis and the number of reads per gene was normalized to gene length.

### Statistical analysis

The Shapiro-Wilk test was used to test normality and correlations between alpha diversity and quantity of extracted DNA (i.e., prior to genome amplification of select samples) were tested using Spearman’s rank order correlation analysis in R. To determine if community composition among samples based on the 16S and 18S rRNA gene data were significantly different (α = 0.05), Adonis tests were run using Vegan in R. The results from MANOVA tests and *post hoc* analysis with Tukey’s Honest Significant Difference test were used to determine statistical significance of relative abundances of taxa among sample types. To examine correlations in the relative abundances of bacteria, fungi, and algae, a Pearson correlation analysis was done in R using the “Hmisc” package and visualized using the “corrplot” package. ANOVA tests were used to compare the relative abundance of genes assigned to bacteria, algae, and fungi among sample types. The Wald test was used to determine statistically significant differences in the abundance of OTUs and functional genes with the DESEQ2 v1.40.1 tool (Love et al., 2014).

## Results

### Metagenomic sequencing output

**Table 1** summarizes the sequencing results from 39 DNA samples extracted from a supraglacial stream (2 samples), meltwater from the WCA (9 samples), near-surface ice (15 samples from depths of 0 to 5m below the surface), and englacial ice (13 samples from depths of 5 to 15 m; **Figure 1**). A total of 174 million paired end (2 × 150 bp) reads were generated, which corresponded to an average of 4 million reads per sample (range of ~75,000 to 60 million reads per sample; **Supplementary Table S1**). Most sequence reads classify as bacterial or eukaryotic (**Table 1**), with < 0.01% of the reads (< 0.1% of total genes) identified as archaeal (i.e. taxa within the Methanococci, Methanomicrobia, and Thermococcales). From these data, 10 million contigs were assembled with a maximum length of 372,000 bp (**Supplementary Table S2**). Attempts at *de novo* assembly for each sample failed to generate a large number of high-quality metagenome-assembled genomes; only 8 bins were obtained with completeness > 80% and contamination < 10%. Presumably, this was due to the short length of the contigs used for the assembly and low coverage for many of the libraries (**Supplementary Table S3**). Therefore, we restricted further analysis to the unassembled reads and contigs containing protein-encoding genes.

**Table 1:** Comparison of basic data outputs from the DNA sequencing and phylogenetic binning of reads from each sample type.

	Supraglacial Stream	Near-surface Ice (0-5m)	Weathering Crust Aquifer	Englacial Ice (5-15m)
Number of samples	2	15	9	13
Reads per sample	1295808	5291961 ± 14523424	3524928 ± 3100957	4385548 ± 5230017
Total number of reads	2591612	79379413	31724351	53792038
% Bacteria	89.2	80.7 ± 22.4	73.9 ± 23.2	86.2 ± 16.7
% Fungi	2.9	6.3 ± 12.5	14.6 ± 19	1 ± 0.5
% Algae	0.6	0.6 ± 0.4	1 ± 0.7	0.3 ± 0.2
% Other Eukarya	1.5	6.6 ± 10.8	4.5 ± 5.9	4.7 ± 6.3
Number of contigs	220861	308167 ± 661535	262737 ± 170151	199043 ± 179982

Values are averages ± the standard deviation of the mean.

### Community composition based on small subunit rRNA sequences in the metagenomes

OTUs were assigned in QIIME2 for the 32 samples that contained > 100 SSU rRNA gene sequences. For 16S rRNA genes, 5,252 bacterial OTUs and 0 archaeal OTUs were generated from a total of 104,932 total reads, whereas fewer eukaryotic 18S rRNA genes (69,421 reads) and OTUs (1,770) were observed (**Supplementary Table S3**). To assess this approach, we analyzed sequence data obtained using samples of the ZymoBIOMICS™ Microbial Community Standard (Catalog No. D6300) and found that the analysis in QIIME produced genus and phylum level predictions that agreed well with the species abundances in the standard (**Supplementary Figure S1**).

Simpson’s complementary diversity (i.e., 1-D) indices for bacteria are high and range from 0.98 to 0.99, with the highest diversity observed in the stream samples (average of 0.988; **Supplementary Table S3**). The Good’s coverage values of species richness for the 16S rRNA gene sequences were lowest in supraglacial samples (average of 0.34) and higher coverage was observed in the WCA (0.18 to 0.81), near-surface ice (0.18 to 0.90), and englacial ice (0.33 to 0.95) samples (**Supplementary Table S3**). This coverage is much lower than values > 0.96 based on amplicon data from these samples (Christner et al., 2018). From analysis of the 18S rRNA gene data, a lower diversity of eukaryotes was indicated by Simpson’s complementary diversity values of 0.81 to 0.99. While Good’s coverage for the 18S and 16S rRNA gene data ranged widely (0.125 to 0.95), the values are not statistically different (ANOVA, p > 0.05) among sample types. Good’s coverage (**Supplementary Table S3**) is moderately positively correlated with the quantity of DNA used to prepare the libraries (Spearman’s r = 0.431, p < 0.01, n = 39), consistent with low coverage being due to the lower amounts of extractable DNA in the samples. The trimmed sequence count was positively correlated with Good’s coverage values for 16S and 18S rRNA genes (r > 0.6, p < 0.001; **Supplementary Table S1**), but did not significantly correlate to Simpson’s complementary diversity. Because we analyzed non-overlapping rRNA gene sequences from the metagenomes, the number of unique OTUs and singletons is likely to be artificially inflated, also contributing to low Good’s coverage values.

**Figure 2:**
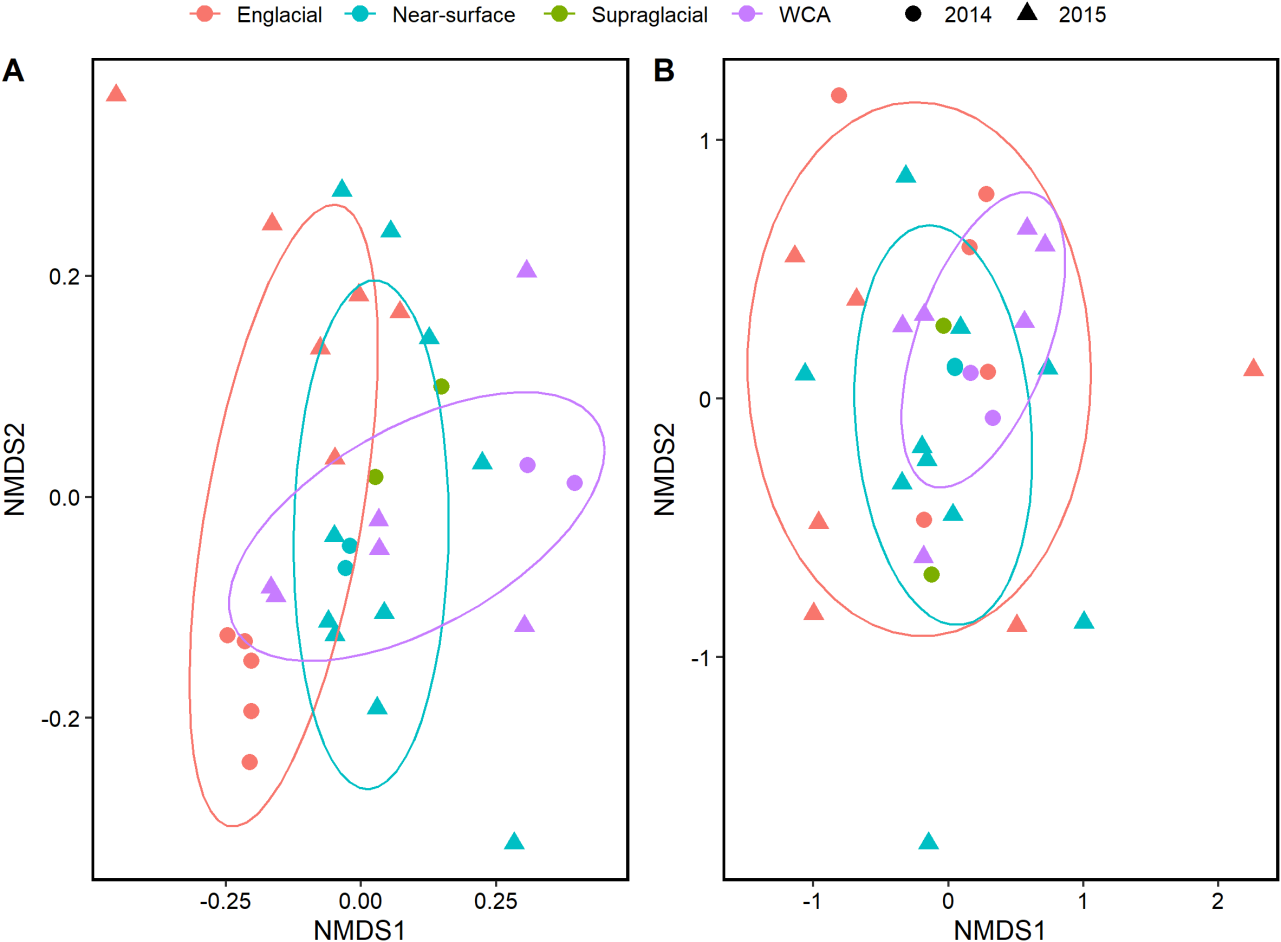
NMDS of Bray-Curtis distance among samples collected during 2014 and 2015 from the supraglacial stream, WCA, and ice from the near-surface and englacial zone. Similarity calculations and statistical significance were performed using Adonis with the vegan package in R. The ellipses indicate the clustering of each sample type with a confidence level of 0.70. **(A)** Analysis based on 16S rRNA genes (stress=0.2209). **(B)** Analysis based on 18S rRNA genes (stress=0.1594). The plots were generated using ggplot2 in R.

Bacterial beta diversity was visualized in NMDS plots of Bray-Curtis dissimilarity distances based on 16S rRNA genes extracted from metagenomic data (**Figure 2A**) and compared with data derived from amplicon sequencing (Christner et al., 2018; **Supplementary Figure S2**). The two-dimensional NMDS ordination for bacteria showed similar clustering of samples between methods and differentiated englacial samples from those of the supraglacial stream, WCA, and near-surface ice (**Figure 2A**), but it has a relatively high ordination stress value of 0.2209. Therefore, we also conducted a PCoA analysis of the 16S rRNA gene data (data not shown), which resulted in a similar clustering of samples. Bacterial assemblages in englacial ice are significantly different from the other samples (p value < 0.05, Adonis), as was also found for amplicon data by Christner et al. (2018). Most of the bacterial community samples from the WCA are most similar to those observed in bulk sampling of near-surface ice. This is not entirely unexpected because the meltwater sampled from the near-surface ice would contain a mixture of taxa originating from the WCA and from the melting of englacial ice. Bacterial community structure in the WCA was highly heterogenous, particularly in the samples collected during three consecutive days in 2015. The NMDS ordination for the 18S rRNA gene data resulted in a lower stress value of 0.1594 and showed compositions that were relatively heterogeneous within themselves and not well differentiated by environmental type. The only significant differences in composition among the eukaryotic communities were between sample years (p value < 0.05, Adonis; **Figure 2B**).

**Figure 3:**
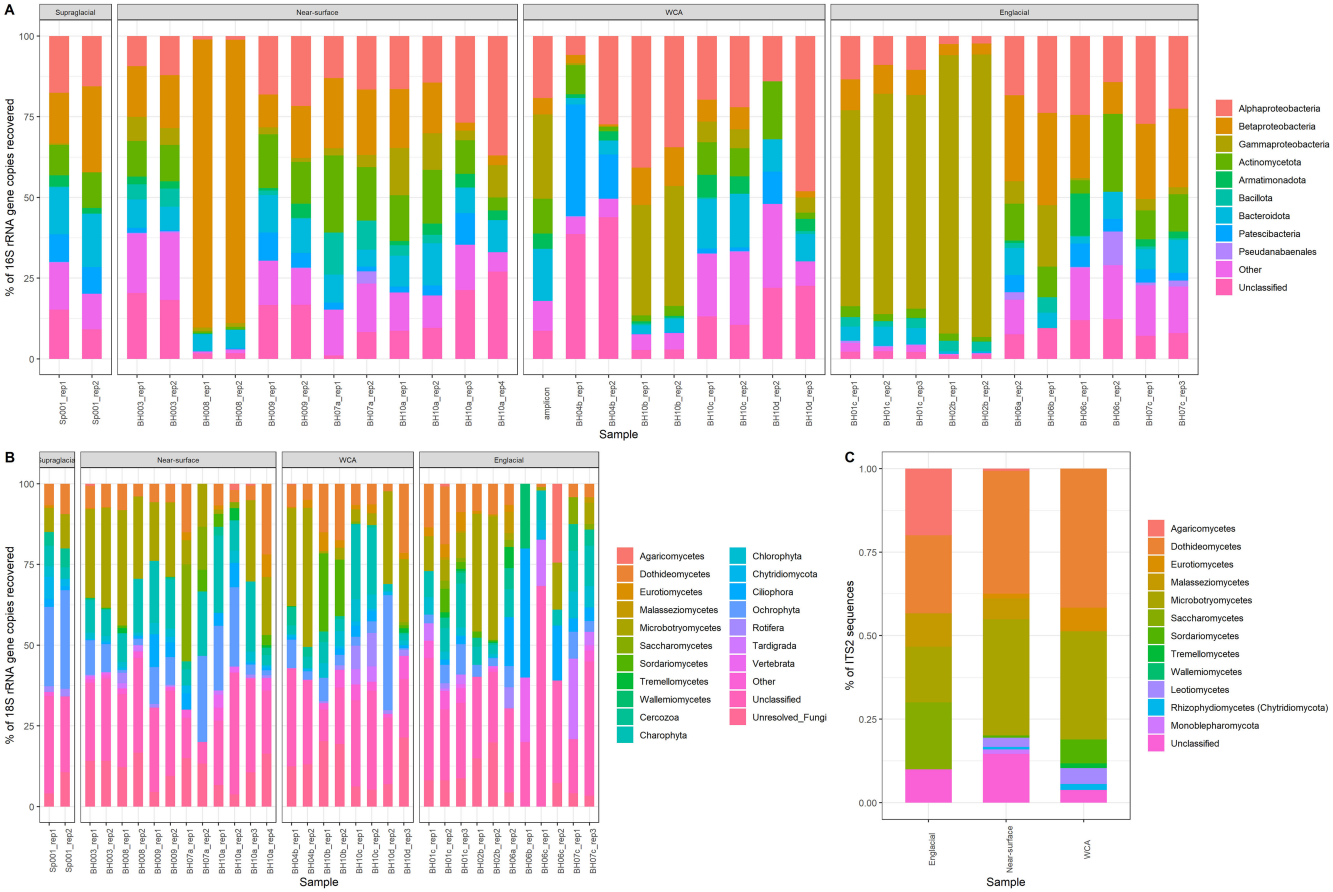
Community composition based on SSU rRNA genes and ITS2 sequences retrieved from metagenomes. **(A)** Relative abundances of bacteria phyla based on 16S rRNA genes, except for Pseudomonadota, which is shown at the class level, and Cyanobacteriota, which is shown at the order level. **(B)** Relative abundances of eukaryotic groups based on 18S rRNA genes. The “Other” category contains taxa with abundances <0.01%. **(C)** Relative abundance of fungal classes based on the internal transcribed spacer 2 (ITS2) sequence.

Pseudomonadota is the most abundant bacterial phylum in the samples (9.9 to 93.5% relative abundance; **Figure 3A**), with most taxa affiliated with class Betaproteobacteria, except for the englacial samples from 2014 in which Gammaproteobacteria were more numerous, similar to results from 16S rRNA gene amplicon sequencing (Christner et al., 2018). The 20 most abundant bacterial OTUs (**Supplementary Table S4**) are Pseudomonadota, and 12 of these classify within the genus *Pseudomonas*. Actinomycetota (0.7 to 24%), Bacteroidota (0.4 to 17%), and Patescibacteria (0 to 35%) were found at high abundances in the majority of samples, and Cyanobacteriota (0 to 25%) was found in several near-surface ice (BH07a_rep1 and BH003_rep2) and englacial ice (BH06b_rep1, BH06c_rep2, and BH07c_rep3) samples (**Figure 3A**). While many of the same bacterial phyla were shared and found in similar abundances among the stream, near-surface ice, WCA, and englacial ice samples, compositional differences were observed for each environmental type. For instance, there are significantly more taxa from the phylum Deinococcota (31 OTUs) in the supraglacial stream samples compared to those from the WCA (MANOVA, p < 0.01, n = 2). The number of gammaproteobacterial 16S rRNA gene reads (807 OTUs) varied significantly among samples (p value < 0.05, MANOVA), with higher numbers of taxa in the WCA than in englacial ice. DESEQ2 analysis showed that 15 and 8 OTUs classifying within the genera *Brevundimonas* and *Stenotrophomonas*, respectively, had significantly higher abundances in the WCA as compared to other samples (**Supplementary Table S5**).

Fungi and unicellular algae were the numerically dominant taxa in the eukaryotic communities, representing 1% to 15% and 0.3% to 1%, respectively, of the total sequencing reads (**Table 1**). The 20 most abundant OTUs are members of Ascomycota, Basidiomycota, Charophyta, and Ochrophyta divisions. Within the Basidiomycota (1 to 57% of the 18S rRNA sequences), OTUs in class Microbotryomycetes are most abundant, followed by Agaricomycetes, Malasseziomycetes, and Wallemiomycetes (**Figure 3B**). Ascomycota was the second most abundant fungal phylum (0 to 64% of the 18S rRNA sequences) and contained OTUs belonging to Dothideomycetes, Eurotiomycetes, Saccharomycetes, Sordariomycetes, and Tremellomycetes (**Figure 3B**). The distribution of fungal classes varies amongst individual samples, but abundance is not significantly different based on sample type. For example, Saccharomycetes was the most abundant group in the near-surface sample BH07a_rep2, with a relatively low abundances across the remainder of samples. Similarly, Sordariomycetes dominated two WCA samples (BH10b_rep1 and BH10b_rep2) but was found at lower abundance in the other WCA samples. The abundance of OTUs from the phylum Chytridiomycota was highest in the supraglacial sample Sp001-rep1 (6.8%). Tardigrada, and Rotifera phyla were present in some of the samples (0% to 25% and 0% to 10%, respectively). Vertebrata were also present in low abundances (0.36% average relative abundance), with BH10a_rep1 containing 4% relative abundance, and the lowest classification assigned to the OTUs is the class Mammalia.

**Figure 4:**
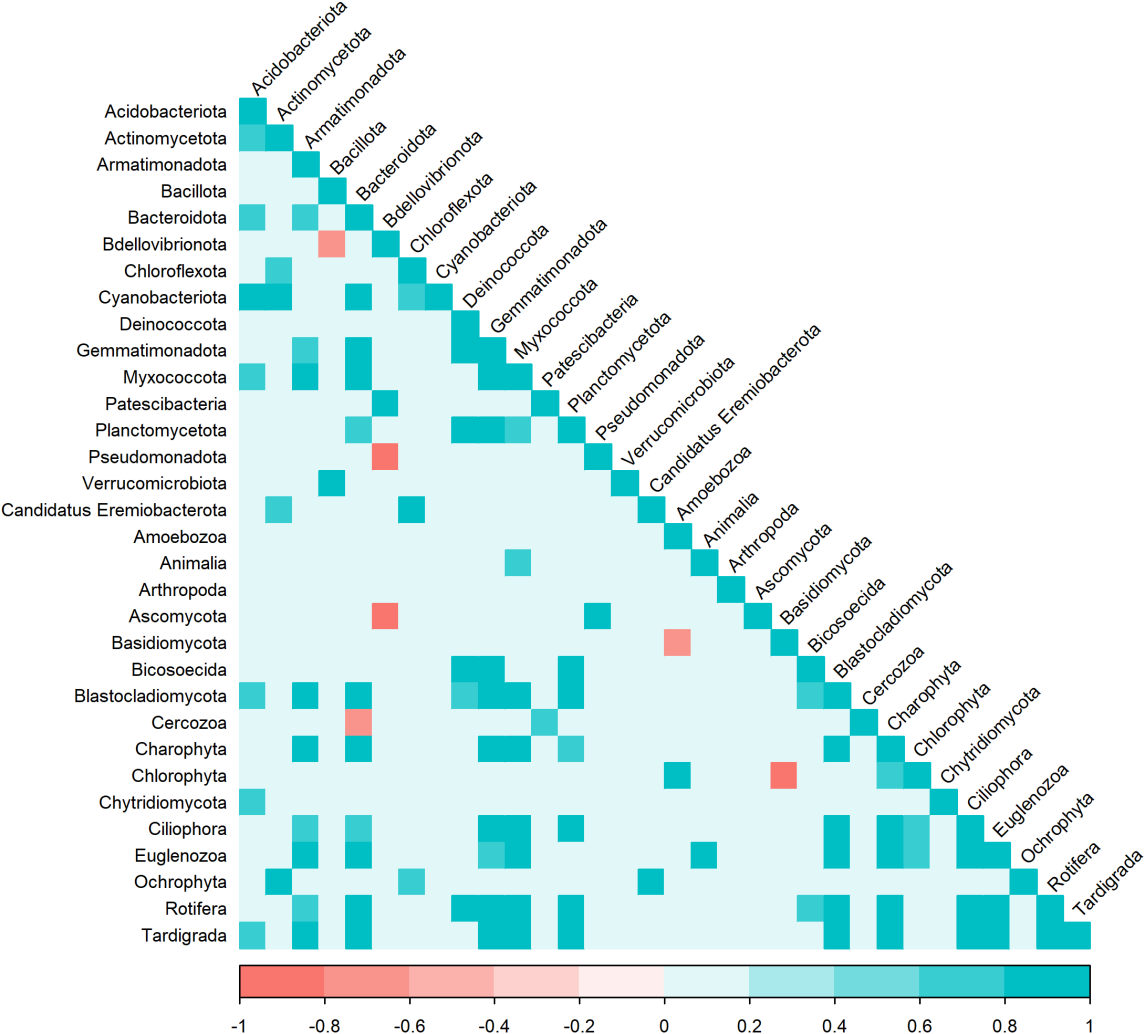
Correlation of bacterial (phylum and class) and eukaryotic (phylum and divisions) SSU rRNA phylotypes in WCA samples. The heat plot was generated based on Pearson correlation in the “Hmisc” package. Statistically different (p < 0.05) and highly correlated variables are shown for groups with greater than 0.5% abundance in the samples.

Certain algae were highly abundant members of the eukaryotic communities, including green (Charophyta and Chlorophyta; 0%-24% and 0%-8%, respectively, of the 18S rRNA gene sequences) and golden (Ochrophyta; 0%-36%) algae. Samples from the supraglacial stream contained significantly more 18S rRNA gene reads for Ochrophyta (MANOVA, p < 0.01, n = 2; 39%-41%) than other sample types (near-surface: 0%-36%; WCA: 1%-41%; englacial ice: 0%-16%; **Figure 3B**). A comparison of bacterial and eukaryotic phylotypes in the WCA samples identified algal divisions with abundances highly positively correlated (r > 0.7, p < 0.05) to eight bacterial phyla or classes (**Figure 4**). Charophyta are positively correlated to Rotifera, Tardigrada, and five bacterial phyla (Armatimonadota, Bacteroidota, Gemmatimonadota, Myxococcota, and Planctomycetota). The relative abundance of Chlorophyta is negatively correlated to Basidiomycota, whereas Ochryophyta positively correlates to three bacterial phyla (Actinomycetota, Chloroflexota, *Candidatus* Eremiobacterota; **Figure 4**). The correlations between relative abundances of specific taxa to major algal groups was also supported by analyses conducted at lower taxonomic hierarchies. OTUs in Zygnematophyceae and Chrysophyceae positively correlate with those in the bacterial phyla Actinomycetota, Parcubacteria, and Pseudomonata (**Table 2**). In addition, Zygnematophyceae positively correlates with 4 OTUs in three bacterial phyla (Armatimonadota, Bacteroidota, and Cyanobacteriota), whereas Chrysophyceae negatively correlates to an OTU in the Armatimonadota phyla. There is also a positive correlation among OTUs belonging to the Zygnematophyceae and Chrysophyceae classes and several groups of fungi (i.e. Ascomycota and Basidiomycota; **Table 2**).

**Table 2:** Number of OTUs correlated to the relative abundance of major algal taxa in samples from the WCA.

Description	Correlation with Zygnematophyceae	Correlation with Chrysophyceae
Actinomycetia (Actinomycetota)	3+	1+
Armatimonadia (Armatimonadota)	2+	2-
Cytophagia (Bacteroidota)	3+	0
Cyanobacteriota- unclassified	1+	0
Parcubacteria- unclassified	1+	1+
Alphaproteobacteria (Pseudomonadota)	3+	1+
Betaproteobacteria (Pseudomonadota)	1+	0
Pseudomonadota- unclassified	1+	0
Ascomycota- unclassified	1+	0
Dothideomycetes (Ascomycota)	0	3+
Basidiomycota- unclassified	5+	4+
Microbotryomycetes (Basidiomycota)	10+	15+
Exobasidiomycetes (Basidiomycota)	1+	0
Chlorophyta- unclassified	1+	0
Chlorophyceae (Chlorophyta)	1+	0
Bdelloidea (Rotifera)	1+	0

Statistically significant (p < 0.05) and highly correlated results [r > 0.6 for positive associations (+) and r < -0.6 for negative associations (-)] are shown.

**Figure 5:**
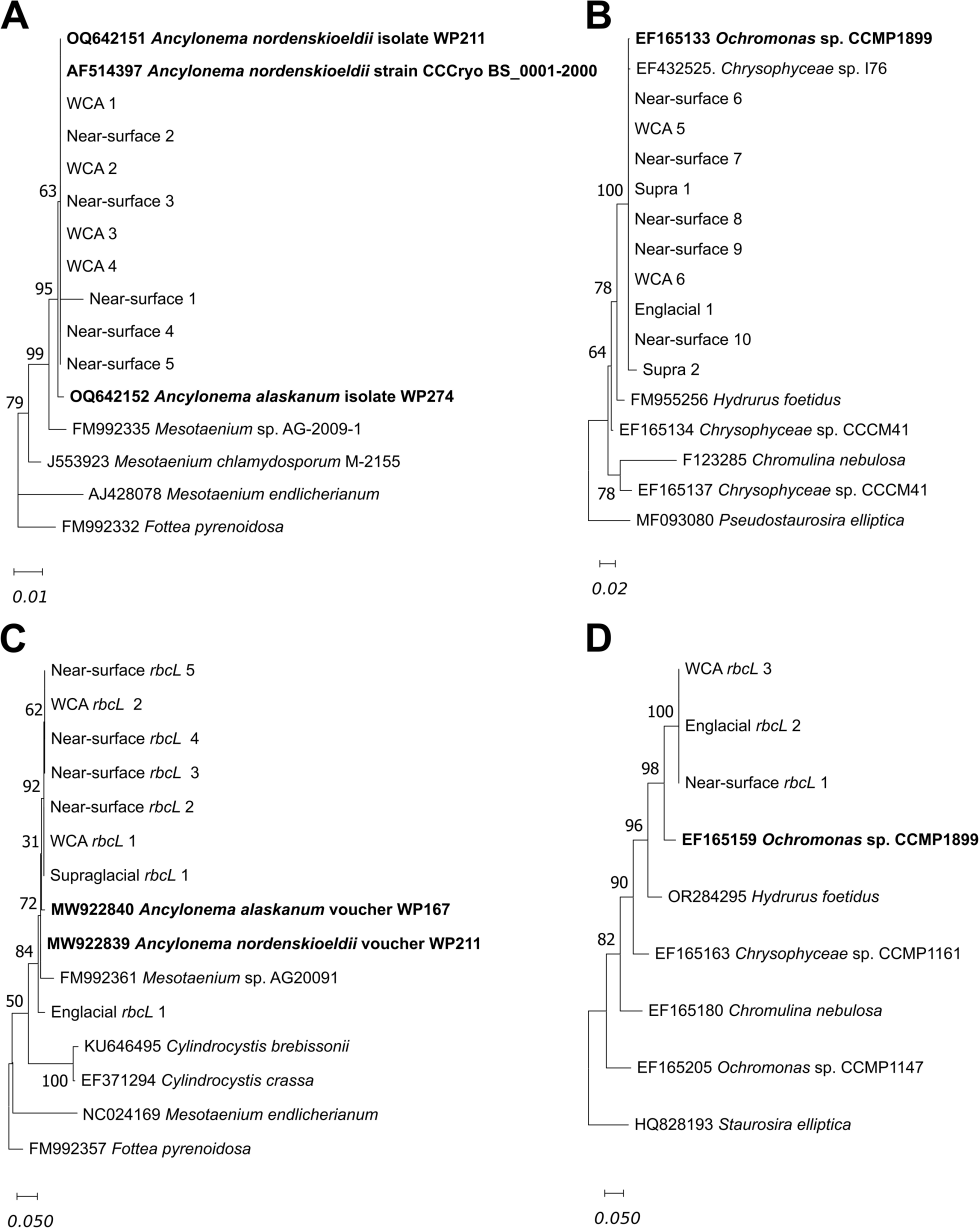
Phylogenetic analysis of partial 18S rRNA (1000 bp ranging from position 271 to 1270; *Fottea pyrenoidosa* FM992332) for Zygnematophyceae **(A)** and Chrysophyceae **(B)** and *rbcL* (387bp) gene sequences for Zygnematophyceae **(C)** and Chrysophyceae **(D)** by maximum likelihood using the Jukes-Cantor model with 100 bootstraps Branches corresponding to partitions reproduced in greater than 30% of bootstrap replicates are indicated. The phylogenetic trees for 18S rRNA and *rbcL* gene analysis were rooted using DNA sequences from *Fottea pyrenoidosa* for sequences belonging to Zygnematophyceae, and *Pseudostaurosira elliptica* for sequences belonging to Chrysophyceae. The scale bar represents the number of changes per nucleotide position. Names in bold are taxa previously reported from ice surfaces.

Analysis of 1000 bp 18S rRNA gene sequences on contigs recovered from the supraglacial stream, WCA, and near-surface ice samples provided information on the phylogeny of organisms within major algal divisions (**Figures 5A, B**). We identified various green and golden algal taxa (9 and 10 sequences, respectively) that are highly similar to those reported in previous studies of snow, glacial ice, and other icy ecosystems. For instance, eight of the 18S rRNA gene sequences are identical to species of *A. nordenskioeldii* that have been documented in supraglacial environments of Svalbard and Switzerland (Leya, 2004; Procházková et al., 2024; **Figure 5A**). A separate cluster of 18S rRNA gene sequences phylogenetically related to golden algae in the genus *Ochromonas* (**Figure 5B**) contains nine OTUs with 99.9% identity to *Ochromonas* CCMP 1899, a mixotrophic golden algal species isolated from Antarctic sea ice (Brodie and Lewis, 2007).

### *rbcL* and ITS2 sequences in the metagenomes

Given the limited phylogenetic resolution provided by the 18S rRNA gene, further analysis of molecular diversity in the eukaryotic community was directed towards analyzing sequences of the internal transcribed spacer 2 (ITS2) and *rbcL* (large subunit of ribulose bisphosphate carboxylase) gene that were identified in the metagenome data. Analysis of 2,568 ITS2 sequences retrieved from the near-surface, WCA, and englacial samples identified 10 classes of fungi (**Figure 5C**) that largely agreed with compositions based on the 18S rRNA gene (15 classes identified; **Figure 5B**), but with notable differences. Both the 18S rRNA gene- and ITS2-based analyses showed that the divisions Ascomycota and Basidiomycota dominated, with the Dothideomycetes and Microbotryomycetes being the most abundant classes in these divisions, comprising 34% and 28%, respectively, of ITS2 sequences (**Figures 5B, C**). These methods also indicate that Chytridiomycota are in low abundance. However, when comparing relative abundances of 18S rRNA gene sequences to ITS2 sequences, Dothideomycetes was identified at lower abundances in the 18S rRNA gene sequences (15.4% of 18S rRNA gene fungal sequences), while Microbotryomycetes had a similar abundance (30.1% of 18S rRNA gene fungal sequences). In addition, Saccharomycetes, Agaricomycetes, and Malasseziomycetes taxa that were at relatively high abundances (5 to 7%) in the ITS2 sequences were inferred to have very low abundances in the 18S rRNA gene data (< 0.7% of fungal sequences; **Figures 5B, C**).

Analysis of 858 *rbcL* reads showed that most sequences (75.4%) were closely related to taxa in the phototrophic genera *Ancylonema*, *Cylindrocystis*, *Mesotaenium*, and *Ochromonas* (**Supplementary Figure S3**). *Ancylonema* and *Cylindrocystis* were most abundant in the WCA, making up half of the *rbcL* reads in these samples. *Ancylonema* was the most abundant genus in supraglacial, near-surface, and englacial samples based on the distribution of reads for *rbcL* (**Supplementary Figure S3**) and 18S rRNA (**Figure 5B**) genes. Analysis of *rbcL* sequences also confirmed the presence of taxa in phylum Ochrophyta (clade Stramenopile) that were identified from 18S rRNA gene analysis (**Figure 5B**). Of the *rbcL* genes in the supraglacial samples, the most abundant were Ochrophyta taxa (25%), which were at lower abundances in the other samples (6-10%; **Supplementary Figure S3**). Analysis of 387 bp *rbcL* gene sequences from contigs showed the samples contained seven *rbcL* sequences with > 98% identity to that of *A. nordenskioeldii* and *A. alaskanum* (**Figure 5C**). Several *rbcL* sequences in the genus *Ochromonas* were also identified, and based on an operational taxonomic unit definition of > 98% identity for *rbcL* (An et al., 2018), the three sequences with 93% similarity to the *rbcL* gene of *Ochromonas* CCMP 1899 (**Figure 5D**) are likely to be a distinct species from the Antarctic sea ice isolate identified as their nearest neighbor by *rbcL* gene analysis.

### Functional analysis of metagenomes

**Figure 6:**
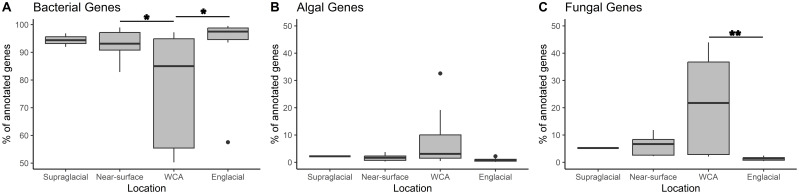
The percentage of genes assigned to bacterial **(A)**, algal **(B)**, and fungal **(C)** taxa for each sampling location. Asterisks show significant differences for *post hoc* analysis of ANOVA results using Tukey’s Honest Significant Difference (*=<0.05, **=<0.01).

Functional annotation of assembled data using DIAMOND-Blastx analysis identified ~1.2 million bacterial and ~70,000 eukaryotic genes (**Supplementary Table S2**). Archaeal functional genes represented less than 0.1% of genes identified in the metagenomes and were excluded from the analysis. Based on sequence analysis conducted using InterPro, the dataset contains 150,796 bacterial and 11,413 eukaryotic protein-encoding genes. Most bacterial genes classify within the groups Actinomycetota, Alphaproteobacteria, Bacteroidota, Cyanobacteriota, and Gammaproteobacteria (**Supplementary Figure S4A**), largely consistent with compositions inferred from the 16S rRNA gene data (**Figure 3A**), with the exception of Patescibacteria, which was found at a much lower abundance (0.1%) compared to 16S rRNA gene data (3.1%). Compositional abundances differed greatly from those inferred from 18S rRNA gene analysis (**Supplementary Figure S4B**; **Figure 3B**). For instance, genes from Ochrophyta were found at very different abundances in the total gene data (18.5%) compared to the 18S rRNA gene data (7.9%), likely due to the lack of reference genomes available. However, Dikarya and Charophyta were found at similar abundances in both datasets. On average, ~90% of the genes identified from the samples were bacterial, but the fraction observed in the WCA samples is lower (76%) and significantly different (p < 0.05) from values in the near surface and englacial ice (**Figure 6A**). The lower fraction of bacterial genes in the WCA is offset by increased abundances of fungal genes (21% compared to <6% for other sites) that are significantly different from the englacial samples (p < 0.01; **Figures 6B, C**). Approximately 3.7% of the genes identified belong to the algal groups Charophyta, Ochrophyta, and Chlorophyta, and although slightly higher percentages of algal genes were observed in the WCA samples (8.5%), the values are not significantly different among locations (**Figure 6B**).

**Figure 7:**
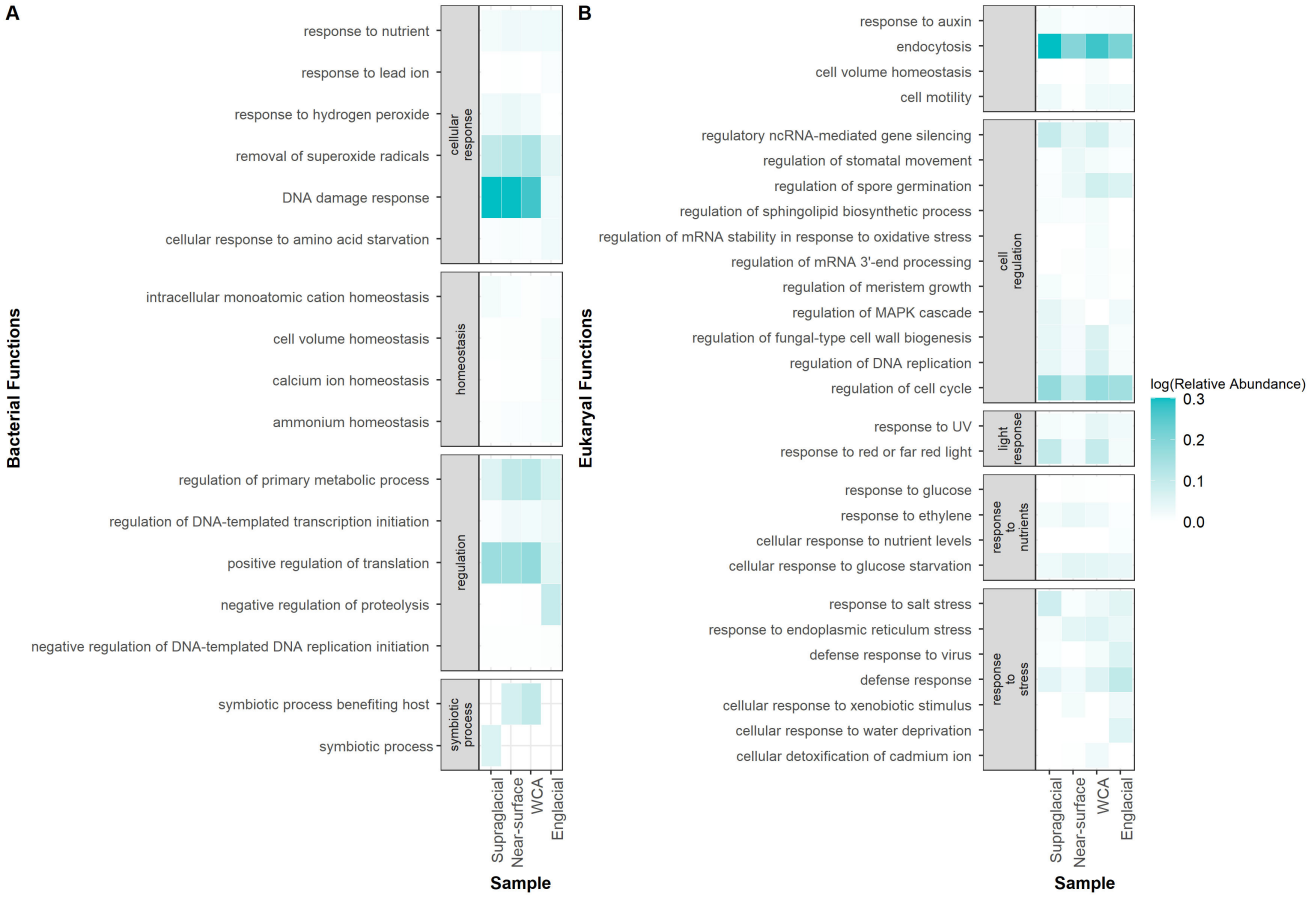
Comparison of GO functions related to stress adaptations significantly enriched or depleted in the WCA (Wald test, p < 0.05). Genes classified as bacterial **(A)** and eukaryotic **(B)** appear in separate panels. Relative abundance of genes was determined by mapping reads to genes, then normalizing by gene length.

DESEQ2 was used to determine if functional genes and Gene Ontology terms (GO terms) were differentially distributed by environmental type (**Supplementary Table S7**). A total of 2,395 bacterial genes (1.6% of total genes) and 984 eukaryotic genes (8.6%) are differentially abundant in the WCA compared to one or more sample types (Wald test, p < 0.05). Bacterial gene functions in the WCA are most distinct from englacial ice, with 85 GO terms differentially abundant, compared to 6 and 13 GO terms when compared to supraglacial and near-surface ice samples, respectively. Eukaryotic gene functions also varied widely between the WCA and the near-surface and englacial ice, with 130 and 148 GO terms differentially abundant, respectively, and that span a wide range of biological processes (**Supplementary Table S7**). Many of the GO functions differentially abundant in the WCA are potentially related to adaptations that enhance environmental fitness and interactions within the ecosystem (**Figure 7**). The functions enriched in the WCA included genes associated with symbiotic interactions (GO:0085030), response to nutrients (GO:0007584), response to salt stress (GO:0009651), and processes that detoxify superoxide radicals (GO:0019430), whereas genes involved in substrate transport were depleted relative to englacial ice (**Supplementary Table S7**). Gene functions for response to UV (GO:0009411) and absence of light (GO:0009646) are also enriched in the WCA compared to near-surface and englacial ice (**Figure 7B**). Curiously, genes involved in response to DNA damage (GO:0006974) are depleted in the WCA compared to other sample types (**Figure 7A**). Gene content of assemblages in the WCA samples were also depleted for cellular response to water deprivation (GO:0042631) and nutrient levels (GO:0031669) when compared to englacial ice.

Genes encoding enzymes involved in carbon, energy, and nutrient metabolism provided insight on the major catabolic and anabolic pathways distributed among the communities (**Supplementary Table S6**). Samples containing genes annotated for photosystem I and II showed the potential for oxygenic photosynthetic pathways (GO:0015979). The presence of the Calvin–Bassham–Benson cycle was indicated by the presence of genes for ribulose bisphosphate carboxylase complex assembly (GO:0110102) and phosphofructokinase-1. Many sample types contained genes associated with lithotrophic metabolisms, including the oxidation of ferrous iron (GO:0019411) and reduced sulfur (GO:0019417; GO:0019418). Samples containing genes for carbohydrate metabolic processing (GO:0005975) coupled with respiration of O_2_ and nitrate were observed, including aerobic respiration (GO:0009060), anaerobic respiration (GO:0009061), and denitrification [GO:0019333]. Metagenomes also encoded enzymes for fermentation via the Embden-Meyerhof (GO:0006096) and Entner-Doudoroff (GO:0009255) pathways. Genes involved in nitrogen fixation (GO:0009399) provided evidence for the potential for diazotrophy in the bacterial communities. Bacterial and fungal genes related to inorganic phosphorus mobilization were also widely distributed among samples, including quinoprotein glucose dehydrogenase A, exopolyphosphatase, inorganic pyrophosphatase, and alkaline phosphatase D (**Supplementary Figure S5**).

Genes involved in response to stresses associated with temperature and light were identified in the supraglacial and englacial assemblages. These included stress related adaptations such as extracellular polysaccharide biosynthetic processes (GO:0045226), response to oxidative (GO:0034599) and osmotic stress (GO:0071470), and carotenoid biosynthesis (GO:0016117; **Supplementary Table S6**). Additionally, genes involved in fungal sporulation and formation of a cellular spore (GO:0030435) were found, as well as genes known to be upregulated in mixotrophic algae (i.e., McKie-Krisberg et al., 2018) and involved in processes such as endocytosis (GO:0006897), phagocytosis (GO:0006909), and autophagy (GO:0006914; **Supplementary Table S6**).

## Discussion

The interplay among physical, hydrological, and microbiological processes in the ablating surfaces of glaciers is recognized as an important research area due to their effects on altering ice properties, meltwater generation, supraglacial nutrient cycling, and the solute composition of runoff (Wadham et al., 2016; Musilova et al., 2017; Williamson et al., 2020). Recent studies of microbial communities inhabiting cryoconite holes, surficial ice, snow, meltwater lakes, and streams have highlighted the diversity of species and metabolisms in glacier and ice sheet microbiomes globally (e.g., Cameron et al., 2016; Anesio et al., 2017; Jaarsma et al., 2023). Bacterial communities in cryoconite holes and surface ice vary by geographical location, yet are largely comprised of taxa from the same phyla we have documented (i.e., Cyanobacteriota, Pseudomonadota, Actinomycetota, and Bacteroidota; Stibal et al., 2015; Cameron et al., 2016) in supraglacial features and englacial ice of a temperate Alaskan glacier (**Figure 3A**). Though few studies have examined microbial assemblages in the near-surface, water-saturated habitats of porous glacial ice, the unique distributions of bacterial and algal taxa reported in the weathering crust environment (Christner et al., 2018; Rassner et al., 2024) support a proposition for communities with distinct functions and biogeochemical roles in supraglacial meta-ecosystems. Hence, the aim of our study was to further understanding of microorganisms in the WCA by comparing their gene contents to communities associated with proximal features of the supraglacial ecosystem.

Low biomass and yields of extractable DNA (i.e., 20 samples with < 1 ng of DNA available for sequencing) produced a metagenomic dataset that assembled into few high-quality bins. While this prevented a broad comparison of metagenome-assembled genomes from the populations, the data available nevertheless allowed us to identify orthologous genes assembled from paired-end sequencing of samples from the WCA to infer molecular function. In comparison to the number of bacterial genes identified (~1,200,000), fewer eukaryotic genes were identified in the samples (~70,000; **Supplementary Table S2**). The 16S rRNA gene sequences recovered from the metagenomes indicate the bacterial communities of the WCA were distinct from samples in the englacial environment (**Figures 2A**, **3A**), whereas analysis of 18S rRNA gene sequences showed that the eukaryotic community composition was similar amongst all sample types (**Figures 2B**, **3B**). Despite the compositional similarities in the eukaryotic communities, we found higher numbers of fungal genes in the WCA in comparison to the other samples analyzed (**Figure 6**), implying that fungi are an important component of the WCA biodiversity and may be playing crucial roles in supraglacial ecosystem processes. Given that subtle differences in environmental conditions and resource availability have evolved ecologically specialized microbial variants over environmental gradients (e.g., Ward et al., 2006; Chase et al., 2018), we analyzed gene content to seek evidence that the supraglacial environment harbors populations with distinct ecologies related to exposure and access to meltwater. The parameters expected to affect distributions of microbes in the WCA are related to hydrological (i.e., retention of water, microbes, and nutrients; flowthrough velocities 4- to 6-orders of magnitude lower than surface streams; Irvine-Fynn et al., 2021) and physical properties (attenuated flux of solar radiation; Christner et al., 2018) of the porous ice. Considering differences in beta diversity among the bacterial communities (**Figure 2A**), it was surprising to find few differences in gene content (**Figure 7A**), suggesting that the supraglacial bacterial communities may have been functionally homogenous. This contrasted with the abundance of functionally annotated eukaryotic genes, which was significantly different among the WCA assemblages (**Figure 7B**). Eukaryotic communities of the WCA were functionally distinct from near-surface ice, as well as englacial ice, and their metagenomes are enriched in genes related to light stress, spore germination, and endocytosis (**Supplementary Table S7B**); properties that may assist WCA microbes in surviving the stresses associated with the low nutrient and temperature conditions on glacier surfaces.

The range of phototrophic and heterotrophic metabolisms we documented in the Matanuska Glacier’s supraglacial environment are similar to those observed previously on glacial surfaces (Margesin and Collins, 2019; **Supplementary Table S6**). Supraglacial communities are exposed to high fluxes of incident solar radiation in the summer, complete darkness and low temperatures during winter, recurring freezing and thawing events, and oligotrophic conditions. Accordingly, genes with functions related to light-induced stress were enriched in the WCA, including those involved in the response to UV radiation exposure, red and far-red light, and oxidative stress (**Figures 7A, B**). Abundance of genes involved in endospore and fungal sporulation in the samples (**Supplementary Table S6**) implies that some species may form these environmentally durable structures to survive unfavorable conditions and disperse between supraglacial features. Cellular survival during freezing and thawing is enhanced by controlling water efflux and osmolyte accumulation (Doyle et al., 2011), and genes related to cell volume homeostasis, response to salt stress, and cellular response to water deprivation were enriched in the WCA samples (**Figures 7A, B**). Though supraglacial ecosystems typically contain low amounts of nutrients (Holland et al., 2019), a sufficient flux of PAR through the ice surface (Christner et al., 2018) coupled with the presence of phototrophic algae (**Figure 5**) is likely to be a key source of organic carbon in the WCA.

Understanding the interactions between photosynthetic primary producers and heterotrophic microbes is important for deciphering carbon cycling and nutrient exchange in the supraglacial environment. On ice surfaces, measured rates of net primary productivity exceed bacterial production (Williamson et al., 2018), implying that algal photosynthate is an essential organic carbon source for heterotrophic bacteria and fungi. We used species abundance correlations based on protein-encoding and rRNA genes to infer potential interactions between taxa identified in the metagenomes. The relative abundance of algal classes Zygnematophyceae and Chrysophyceae in the WCA were positively correlated to select bacterial taxa (i.e. Actinomycetota, Armatimonadota, Bacteroidota, Cyanobacteriota, and Pseudomonadota) (**Figure 4**; **Table 2**), similar to previous observations on ice surfaces (Nicholes et al., 2019). This result is supported by co-cultivation experiments that have demonstrated cross feeding between snow algae and bacteria (Krug et al., 2020) and that have indicated the algal community is also highly codependent on the bacteria they coexist with. For instance, most algal species lack the genes to produce B vitamins and must rely on the supply of this essential cofactor from bacteria (Croft et al., 2005; Helliwell et al., 2011). Members of the Bacteroidota phylum have previously been shown to be associated with Zygnematophyceae taxa and express genes for the production of thiamine, cobalamin, and biotin in the presence of these algae (Krohn-Molt et al., 2017). Given that bacterial genes for cobalamin (B_12_) biosynthesis are found across all sample types (**Supplementary Table S6**), mutualistic relationships between supraglacial algae and bacteria based on exchange of organic carbon and B_12_ is a plausible ecological scenario. Importantly, if these bacteria are involved in interactions that provide a nutrient essential for algal growth, then they would have key roles in the occurrence of algal blooms on ice surfaces (Yallop et al., 2012; Williamson et al., 2020) and dynamics of supraglacial carbon cycling.

Phosphorus availability has been shown to limit primary productivity in algal blooms on the Greenland Ice Sheet (McCutcheon et al., 2021), calling attention to the sources of this key nutrient in glacial meltwaters. The mineralization of organic phosphorus compounds by fungi (e.g., *Penicillium* (Eurotiomycetes); **Figure 3B**, Qiao et al., 2019) may represent an important source of inorganic phosphorus to the supraglacial environment. The presence of fungal genes encoding inorganic pyrophosphatase in the metagenomes, which hydrolyzes polyphosphate compounds (**Supplementary Figure S5**; Nannipieri et al., 2010), and bacteria known to be efficient at solubilizing phosphate from mineral sources (e.g., *Massilia*; Zheng et al., 2017) supports this contention. Positive correlations were noted between several algal and fungal taxa (OTUs in the Microbotryomycetes and unclassified Basidiomycota; **Table 2**), indicating that fungal taxa may support the growth of algae. Positive correlations between Alphaproteobacteria and several algal taxa (**Table 2**) also imply that bacteria may be important for providing phosphorus to algae, as several alphaproteobacterial phosphorus cycling genes were identified (**Supplementary Figure S5**). We also found positive associations between Zygnematophyceae and Cytophagia (**Table 2**), which is a group of bacteria known to be involved in phosphorus solubilization in soils and predicted to increase phosphate bioavailability (Wu et al., 2022). Fungi isolated from glacial ice have been shown to produce antibacterial secondary metabolites (de Menezes et al., 2020), and many of our samples contained genes encoding pathways for production of mycophenolic acid, polyketides, and alkaloids belonging to Eurotiomycetes and Dothideomycetes, which are abundant in WCA samples (**Figure 3C**; **Supplementary Table S6**). If derivatives of these compounds are produced *in situ*, these secondary metabolites could be involved in antagonistic interactions between supraglacial fungal and bacterial communities that affect community composition and carbon cycling.

Glacial ice surfaces are well known to be colonized by species of green algae from the class Zygnematophyceae that include *Cyclindrocystis brebbisonni*, *A. nordenskioeldii*, and *A. alaskanum* (Yallop et al., 2012; Procházková et al., 2021). *Ancylonema* has been documented on the surfaces of glaciers across the northern hemisphere (Procházková et al., 2021; Takeuchi et al., 2003, 2019; Yallop et al., 2012; Yoshimura et al., 1997), with fewer observations in the southern hemisphere (e.g., maritime Antarctica; Komárek and Komárek, 2001; Ling and Seppelt, 1990). However, since many of these studies used classical morphological approaches as the basis for identification, it is not possible to evaluate their relatedness to the taxa documented in this study. Given that blooms of darkly pigmented *Ancylonema* have been shown to reduce ice albedo (Yallop et al., 2012; Williamson et al., 2020), high abundances of these algae in water-saturated ice on the surface may accelerate melting as well (Cook et al., 2020). The near-surface ice and WCA samples contained high abundances of taxa with 100% identity to the 18S rRNA gene of isolate *A. nordenskioeldii* WP211 from Svalbard/Switzerland (Procházková et al., 2024; OQ642151) and > 99% identity to the *rbcL* genes of *A. nordenskioeldii* WP211 and *A. alaskanum* WP167 (MW922839 and MW922840; Procházková et al., 2021; **Figures 5A, C**) from Austria and Switzerland, respectively. These results indicate there is a high degree of relatedness in green algae separated by large geographical distances, suggesting the possibility of dispersal via atmospheric transport, similar to distribution patterns in certain red-snow algae (Segawa et al., 2018). Although aerial dissemination has been documented for many microorganisms, little is known about the environmental barriers limiting dispersal of ice algae on regional to global scales. Long distance dissemination via air transport and fall-out with dry or wet deposition is possible and supported by the high similarities of our data to phylotypes from distant locations. Future efforts aimed at recovering metagenomic assembled genomes or single amplified genomes from *Ancylonema* populations in supraglacial environments from different geographical locations represent fertile territory for testing the concept of ecological cosmopolitanism in these glacier algae. Due to their role in both carbon cycling and meltwater production in surface ice (Cook et al., 2020; Musilova et al., 2017), understanding the scale of ice algae dispersal is critical for predicting changes to glacial surfaces in the future.

Algae phylogenetically related to an *Ochromonas* species (CCMP1899) isolated from Antarctic sea ice (18S rRNA and *rbcL* genes with > 99.9 and 93% identity, respectively) were also identified (**Figures 5B, D**). While members from this group have been less commonly reported on glacier and ice sheet surfaces than *Ancylonema*, *Ochromonas* taxa are known to be associated with algal blooms in alpine snowpacks (Tanabe et al., 2011) and possess a mixotrophic nutrition based on nutrient and light availability (Lie et al., 2018). Considering their metabolic flexibility, the presence of taxa related to *Ochromonas* in the WCA and near-surface ice samples, and the identification of genes involved in autophagy and endocytosis (**Figure 7**; **Supplementary Table S6**), these microbes may be able to use sunlight or bacterial grazing for energy and nutrition. In the shaded, oligotrophic conditions characteristic of the WCA, the efficiency of a mixotrophic metabolism (e.g., Mitra et al., 2014) could provide golden algae with an advantageous physiological strategy when nutrient and energy sources are limited. In addition to enhancing growth rates and biomass production, mixotrophic microalgae may also be more resilient to the seasonal environmental stresses associated with the ice habitat. While the role of heterotrophic metabolism in algal adaptation to extended period of darkness remains a subject of debate (e.g., Lyon and Mock, 2014), shifts to mixotrophy have been documented in later winter sea ice communities (Bachy et al., 2011). Given the extended persistence of meltwater in the ice (~7.5 months per year; Christner et al., 2018), such a physiological adaptation could extend supraglacial carbon cycling beyond the melt season into periods where decreased solar irradiance and temperature prevent photobiogeochemical processes from occurring on the uppermost ice surface.

As few studies have examined the mycobiomes of glaciers and ice sheets, there is an incomplete understanding of the diversity, ecology, and biogeochemical roles of the fungal communities ubiquitous in supraglacial environments. The fungal groups we identified (**Figures 3B, C**) have also been shown to be associated with surface ice (Microbotryomycetes, Basidiomycota) and snow (Dothideomycetes, Ascomycota and Agaricomycetes, Basidiomycota; Edwards et al., 2013; Brown et al., 2015; Duo Saito et al., 2018; Perini et al., 2019). Several studies have also documented a parasitic relationship between Chytridiomycota and glacier ice algae (Perini et al., 2022; Nakanishi et al., 2023; Kobayashi et al., 2023). The abundance of taxa related to the parasitic Chytridriomycota that infect snow and ice algae (Perini et al., 2022; Nakanishi et al., 2023; Kobayashi et al., 2023) was < 1.6% of total 18S rRNA gene reads (**Figure 3B**). These are distributions similar to those observed in other supraglacial environments (0.01 to 3.4% in surface ice; Perini et al., 2019) where their interactions with glacier algae are inferred to regulate population sizes and affect supraglacial food webs. Microbotryomycetes OTUs and the major algae taxa are positively correlated, and though their specific interactions with microalgae have not been reported, numerous species are known to be phytoparasites of plants (Kemler et al., 2020). In aquatic ecosystems, fungi typically outperform bacteria in decomposition (Gessner et al., 2007) and can often break down complex organic matter not metabolized by bacteria, such as lignin (Sun et al., 2020; Gu et al., 2022). Fungi likely play an important role as decomposers in supraglacial systems. This is supported by observations of high abundances of Microbotromycetes (**Figure 3C**), which are known to be saprotrophic (Oberwinkler, 2017), and fungal genes involved in lignin and pectin catabolism (**Supplementary Table S6**). Fungal interactions that control algal production could impact the biological darkening of ice, but it is also possible that some fungi could have a more direct role in melt water production through the production of pigments that can darken ice surfaces. The fungal metagenomes contain genes for the biosynthesis of melanin (GO:0042438) and carotenoids (GO:0016117; **Supplementary Table S6**) from classes that were highly abundant in supraglacial samples, including Dothideomycetes and Sordariomycetes (**Figure 3C**). These pigments protect cells from UV radiation and lead to increased heat absorption (Avalos and Carmen Limón, 2015; Cordero et al., 2018; Eisenman and Casadevall, 2012). Ice algal pigments have been studied extensively for their role in reducing ice albedo (Remias et al., 2012; Yallop et al., 2012; Williamson et al., 2020), but little is known on the role of fungal or bacterial pigments in ice darkening and meltwater generation in supraglacial environments.

Our analysis of data from two melt seasons verified the hypothesis that the physical and hydrogeochemical conditions in the Matanuska Glacier’s WCA selects for a distinct microbial community. The WCA communities contained a distinct bacterial composition and threefold more fungal genes than those in proximal streams and glacial ice. Although the eukaryotic communities were taxonomically more uniform across the portion of the supraglacial meta-ecosystem we investigated, the gene content of fungi and algae in the WCA are functionally specialized and enriched with genes involved in endocytosis, light stress, and osmotic stress. Additionally, algal abundances in the WCA positively correlated with specific bacterial and fungal taxa that they may interact with to exchange nutrients and carbon sources. The roles of fungal communities in supraglacial biogeochemical processes have been largely ignored to date but are likely to have important implications to nutrient mineralization, production of secondary metabolites, and possibly, contribute to biological darkening of ice. Future studies that trace carbon flow, investigate species interactions, and examine gene expression within the WCA would be valuable for improving understanding of biogeochemical processing in supraglacial microbial communities. Such information would aid scientists with predicting changes in proglacial nutrient export as rates of global temperature and marginal ablation increase.

## Data Availability

The original contributions presented in the study are publicly available. This data can be found here: NCBI SRA, accession PRJNA430887.

## References

[B1] An S. M. Choi D. H. Lee H. Lee J. H. Noh J. H. (2018) Next-generation sequencing reveals the diversity of benthic diatoms in tidal flats. Algae. 33, 167–180.

[B2] Anesio A. M. Hodson A. J. Fritz A. Psenner R. Sattler B. (2009). High microbial activity on glaciers: Importance to the global carbon cycle. Glob. Chang. Biol. 15, 955–960. doi: 10.1111/j.1365-2486.2008.01758.x

[B3] Anesio A. M. Lutz S. Chrismas N. A. M. Benning L. G. (2017). The microbiome of glaciers and ice sheets. NPJ Biofilms. Microbiomes. 3. doi: 10.1038/s41522-017-0019-0 PMC5460203 28649411

[B4] Avalos J. Carmen Limón M. (2015). Biological roles of fungal carotenoids. Curr. Genet. 61, 309–324. doi: 10.1007/s00294-014-0454-x 25284291

[B5] Bachy C. López-García P. Vereshchaka A. Moreira D. (2011). Diversity and vertical distribution of microbial eukaryotes in the snow, sea ice and seawater near the North Pole at the end of the polar night. Front. Microbiol. 2. doi: 10.3389/fmicb.2011.00106 PMC3153057 21833337

[B6] Bairoch A. Apweiler R. (2000). The SWISS-PROT protein sequence database and its supplement TrEMBL in 2000. Nucleic Acids Research, 28(1), 45–48. doi: 10.1093/nar/28.1.45 10592178 PMC102476

[B7] Bankevich A. Nurk S. Antipov D. Gurevich A. A. Dvorkin M. Kulikov A. S.. (2012). SPAdes: A new genome assembly algorithm and its applications to single-cell sequencing. J. Comput. Biol. 19, 455–477. doi: 10.1089/cmb.2012.0021 22506599 PMC3342519

[B8] Bokulich N. A. Kaehler B. D. Rideout J. R. Dillon M. Bolyen E. Knight R.. (2018). Optimizing taxonomic classification of marker-gene amplicon sequences with QIIME 2’s q2-feature-classifier plugin. Microbiome 6. doi: 10.1186/s40168-018-0470-z PMC5956843 29773078

[B9] Bolger A. M. Lohse M. Usadel B. (2014). Trimmomatic: A flexible trimmer for Illumina sequence data. Bioinformatics. 30, 2114–2120. doi: 10.1093/bioinformatics/btu170 24695404 PMC4103590

[B10] Brodie J. Lewis J. (2007). Unravelling the algae: the past, present, and future of algal systematics (Boca Raton: CRC).

[B11] Brown S. P. Olson B. J. S. C. Jumpponen A. (2015). Fungi and algae co-occur in snow: an issue of shared habitat or algal facilitation of heterotrophs? Arct. Antarct. Alp. Res. 47, 729–749. doi: 10.1657/AAAR0014-071

[B12] Buchfink B. Reuter K. Drost H. G. (2021). Sensitive protein alignments at tree-of-life scale using DIAMOND. Nat. Methods 18, 366–368. doi: 10.1038/s41592-021-01101-x 33828273 PMC8026399

[B13] Bushnell B. Rood J. Singer E. (2017). BBMerge – Accurate paired shotgun read merging via overlap. PloS One 12. doi: 10.1371/journal.pone.0185056 PMC5657622 29073143

[B14] Camacho C. Coulouris G. Avagyan V. Ma N. Papadopoulos J. Bealer K.. (2009). BLAST+: architecture and applications. BMC Bioinf. 10. doi: 10.1186/1471-2105-10-421 PMC2803857 20003500

[B15] Cameron K. Stibal M. Zarsky J. D. Gozderelier E. Schostag M. Jacobsen C. S. (2016). Supraglacial Bacterial Communities vary across the Greenland ice sheet. FEMS Microbiol. Ecol. 92, 1–11. doi: 10.1093/femsec/fiv164 26691594

[B16] Caporaso J. G. Kuczynski J. Stombaugh J. Bittinger K. Bushman F. D. Costello E. K.. (2010). QIIME allows analysis of high-throughput community sequencing data. Nat. Methods 7, 335–336. doi: 10.1038/nmeth.f.303 20383131 PMC3156573

[B17] Chase A. B. Gomez-Lunar Z. Lopez A. E. Li J. Allison S. D. Martiny A. C.. (2018). Emergence of soil bacterial ecotypes along a climate gradient. Environ. Microbiol. 20, 4112–4126. doi: 10.1111/1462-2920.14405 30209883

[B18] Christner B. C. Lavender H. F. Davis C. L. Oliver E. E. Neuhaus S. U. Myers K. F.. (2018). Microbial processes in the weathering crust aquifer of a temperate glacier. Cryosphere 12, 3653–3669. doi: 10.5194/tc-12-3653-2018

[B19] Christner B. C. Priscu J. C. Achberger A. M. Barbante C. Carter S. P. Christianson K.. (2014). A microbial ecosystem beneath the West Antarctic ice sheet. Nature 512, 310–313. doi: 10.1038/nature13667 25143114

[B20] Constable A. J. Harper S. Dawson J. Holsman K. Mustonen T. Piepenburg D.. (2023). “Polar regions,” in Climate Change 2022 – Impacts, Adaptation and Vulnerability (Cambridge University Press, Cambridge, UK and New York, NY, USA), 2319–2368. doi: 10.1017/9781009325844.023

[B21] Cook J. Edwards A. Takeuchi N. Irvine-Fynn T. (2016a). Cryoconite: The dark biological secret of the cryosphere. Prog. Phys. Geogr. 40, 66–111. doi: 10.1177/0309133315616574

[B22] Cook J. M. Hodson A. J. Anesio A. M. Hanna E. Yallop M. Stibal M.. (2012). An improved estimate of microbially mediated carbon fluxes from the Greenland ice sheet. J. Glaciol. 58, 1098–1108. doi: 10.3189/2012JoG12J001

[B23] Cook J. M. Hodson A. J. Irvine-Fynn T. D. L. (2016b). Supraglacial weathering crust dynamics inferred from cryoconite hole hydrology. Hydrol. Process. 30, 433–446. doi: 10.1002/hyp.10602

[B24] Cook J. M. Tedstone A. J. Williamson C. Mccutcheon J. Hodson A. J. Dayal A.. (2020). Glacier algae accelerate melt rates on the south-western Greenland Ice Sheet. Cryosphere 14, 309–330. doi: 10.5194/tc-14-309-2020

[B25] Cooper M. G. Smith L. C. Rennermalm A. K. Mige C. Pitcher L. H. Ryan J. C.. (2018). Meltwater storage in low-density near-surface bare ice in the Greenland ice sheet ablation zone. Cryosphere 12, 955–970. doi: 10.5194/tc-12-955-2018

[B26] Cordero R. J. B. Robert V. Cardinali G. Arinze E. S. Thon S. M. Casadevall A. (2018). Impact of yeast pigmentation on heat capture and latitudinal distribution. Curr. Biol. 28, 2657–2664.e3. doi: 10.1016/j.cub.2018.06.034 30078567 PMC6245944

[B27] Croft M. T. Lawrence A. D. Raux-Deery E. Warren M. J. Smith A. G. (2005). Algae acquire vitamin B12 through a symbiotic relationship with bacteria. Nature 438, 90–93. doi: 10.1038/nature04056 16267554

[B28] de Menezes G. C. A. Porto B. A. Amorim S. S. Zani C. L. de Almeida Alves T. M. Junior P. A. S.. (2020). Fungi in glacial ice of Antarctica: diversity, distribution and bioprospecting of bioactive compounds. Extremophiles 24, 367–376. doi: 10.1007/s00792-020-01161-5 32157393

[B29] Doyle S. Dieser M. Broemsen E. Christner B. (2011). “General characteristics of cold-adapted microorganisms,” in Polar Microbiology (ASM Press, Washington, DC, USA), 101–125. doi: 10.1128/9781555817183.ch5

[B30] Duo Saito R. A. Connell L. Rodriguez R. Redman R. Libkind D. de Garcia V. (2018). Metabarcoding analysis of the fungal biodiversity associated with Castaño Overa Glacier – Mount Tronador, Patagonia, Argentina. Fungal Ecol. 36, 8–16. doi: 10.1016/j.funeco.2018.07.006

[B31] Edwards A. Douglas B. Anesio A. M. Rassner S. M. Irvine-Fynn T. D. L. Sattler B.. (2013). A distinctive fungal community inhabiting cryoconite holes on glaciers in Svalbard. Fungal Ecol. 6, 168–176. doi: 10.1016/j.funeco.2012.11.001

[B32] Eisenman H. C. Casadevall A. (2012). Synthesis and assembly of fungal melanin. Appl. Microbiol. Biotechnol. 93, 931–940. doi: 10.1007/s00253-011-3777-2 22173481 PMC4318813

[B33] Fiołka M. J. Takeuchi N. Sofińska-Chmiel W. Wójcik-Mieszawska S. Irvine-Fynn T. Edwards A. (2021). Morphological and spectroscopic analysis of snow and glacier algae and their parasitic fungi on different glaciers of Svalbard. Sci. Rep. 11. doi: 10.1038/s41598-021-01211-8 PMC8575968 34750421

[B34] Foreman C. M. Cory R. M. Morris C. E. Sanclements M. D. Smith H. J. Lisle J. T.. (2013). Microbial growth under humic-free conditions in a supraglacial stream system on the Cotton Glacier, Antarctica. Environ. Res. Lett. 8. doi: 10.1088/1748-9326/8/3/035022

[B35] Gessner M. O. Gulis V. Kuehn K. A. Chauvet E. Suberkropp K. (2007). “Fungal decomposers of plant litter in aquatic systems, ” in Environmental and microbial relationships (Heidelberg: Springer), 301.

[B36] Gu D. Xiang X. Wu Y. Zeng J. Lin X. (2022). Synergy between fungi and bacteria promotes polycyclic aromatic hydrocarbon cometabolism in lignin-amended soil. J. Hazard. Mater. 425. doi: 10.1016/j.jhazmat.2021.127958 34894508

[B37] Heckmann T. Mccoll S. Morche D. (2016). Retreating ice: Research in pro-glacial areas matters. Earth Surf. Process. Landf. 41, 271–276. doi: 10.1002/esp.3858

[B38] Helliwell K. E. Wheeler G. L. Leptos K. C. Goldstein R. E. Smith A. G. (2011). Insights into the evolution of vitamin B 12 auxotrophy from sequenced algal genomes. Mol. Biol. Evol. 28, 2921–2933. doi: 10.1093/molbev/msr124 21551270

[B39] Hodson A. Cameron K. Bøggild C. Irvine-Fynn T. Langford H. Pearce D.. (2010). The structure, biological activity and biogeochemistry of cryoconite aggregates upon an Arctic valley glacier: Longyearbreen, Svalbard. J. Glaciol. 56, 349–362. Available at: https://www.cambridge.org/core.

[B40] Hodson A. Paterson H. Westwood K. Cameron K. Laybourn-Parry J. (2013). A blue-ice ecosystem on the margins of the East Antarctic ice sheet. J. Glaciol. 59, 255–268. doi: 10.3189/2013JoG12J052

[B41] Hoffman M. J. Fountain A. G. Liston G. E. (2014). Near-surface internal melting: A substantial mass loss on Antarctic Dry Valley glaciers. J. Glaciol. 60, 361–374. doi: 10.3189/2014JoG13J095

[B42] Hoham R. W. Duval B. (2001). Microbial Ecology of Snow and Freshwater Ice with Emphasis on Snow Algae. (Cambridge: Cambridge University Press).

[B43] Holland A. T. Williamson C. J. Sgouridis F. Tedstone A. J. McCutcheon J. Cook J. M.. (2019). Dissolved organic nutrients dominate melting surface ice of the Dark Zone (Greenland Ice Sheet). Biogeosciences 16, 3283–3296. doi: 10.5194/bg-16-3283-2019

[B44] Huson D. H. Beier S. Flade I. Górska A. El-Hadidi M. Mitra S.. (2016). MEGAN community edition - interactive exploration and analysis of large-scale microbiome sequencing data. PloS Comput. Biol. 12. doi: 10.1371/journal.pcbi.1004957 PMC4915700 27327495

[B45] Hyatt D. Chen G.-L. Locascio P. F. Land M. L. Larimer F. W. Hauser L. J. (2010). Prodigal: prokaryotic gene recognition and translation initiation site identification. BMC Bioinformatics, 11. doi: 10.1186/1471-2105-11-119 PMC2848648 20211023

[B46] Irvine-Fynn T. D. L. Edwards A. (2014). A frozen asset: The potential of flow cytometry in constraining the glacial biome. Cytomet. Part A. 85, 3–7. doi: 10.1002/cyto.a.22411 24273193

[B47] Irvine-Fynn T. D. L. Edwards A. Newton S. Langford H. Rassner S. M. Telling J.. (2012). Microbial cell budgets of an Arctic glacier surface quantified using flow cytometry. Environ. Microbiol. 14, 2998–3012. doi: 10.1111/j.1462-2920.2012.02876.x 23016868

[B48] Irvine-Fynn T. D. L. Edwards A. Stevens I. T. Mitchell A. C. Bunting P. Box J. E.. (2021). Storage and export of microbial biomass across the western Greenland Ice Sheet. Nat. Commun. 12, 3960. doi: 10.1038/s41467-021-24040-9 34172727 PMC8233322

[B49] Jaarsma A. H. Sipes K. Zervas A. Jiménez F. C. Ellegaard-Jensen L. Thøgersen M. S.. (2023). Exploring microbial diversity in Greenland Ice Sheet supraglacial habitats through culturing-dependent and -independent approaches. FEMS Microbiol. Ecol, 99. doi: 10.1093/femsec/fiad119 PMC10580271 37791411

[B50] Kang D. D. Li F. Kirton E. Thomas A. Egan R. An H.. (2019). MetaBAT 2: An adaptive binning algorithm for robust and efficient genome reconstruction from metagenome assemblies. PeerJ 2019. doi: 10.7717/peerj.7359 PMC6662567 31388474

[B51] Karlstrom L. Zok A. Manga M. (2014). Near-surface permeability in a supraglacial drainage basin on the Llewellyn Glacier, Juneau Icefield, British Columbia. Cryosphere 8, 537–546. doi: 10.5194/tc-8-537-2014

[B52] Kemler M. Denchev T. T. Denchev C. M. Begerow D. Piątek M. Lutz M. (2020). Host preference and sorus location correlate with parasite phylogeny in the smut fungal genus Microbotryum (Basidiomycota, Microbotryales). Mycol. Prog. 19, 481–493. doi: 10.1007/s11557-020-01571-x

[B53] Kobayashi K. Takeuchi N. Kagami M. (2023). High prevalence of parasitic chytrids infection of glacier algae in cryoconite holes in Alaska. Sci. Rep. 13, 3973. doi: 10.1038/s41598-023-30721-w 36894609 PMC9998860

[B54] Komárek O. Komárek J. (2001). Contribution to the taxonomy and ecology of green cryosestic algae in the summer season 1995-96 at Kang George lsland, S. Shetland lslands. Algae. Extreme. Environ. 123, 121–140.

[B55] Krohn-Molt I. Alawi M. Förstner K. U. Wiegandt A. Burkhardt L. Indenbirken D.. (2017). Insights into Microalga and bacteria interactions of selected phycosphere biofilms using metagenomic, transcriptomic, and proteomic approaches. Front. Microbiol. 8. doi: 10.3389/fmicb.2017.01941 PMC5641341 29067007

[B56] Krug L. Erlacher A. Markut K. Berg G. Cernava T. (2020). The microbiome of alpine snow algae shows a specific inter-kingdom connectivity and algae-bacteria interactions with supportive capacities. ISME. J. 14, 2197–2210. doi: 10.1038/s41396-020-0677-4 32424246 PMC7608445

[B57] Kumar S. Stecher G. Li M. Knyaz C. Tamura K. (2018). MEGA X: Molecular evolutionary genetics analysis across computing platforms. Mol. Biol. Evol. 35, 1547–1549. doi: 10.1093/molbev/msy096 29722887 PMC5967553

[B58] Kuraku S. Zmasek C. M. Nishimura O. Katoh K. (2013). aLeaves facilitates on-demand exploration of metazoan gene family trees on MAFFT sequence alignment server with enhanced interactivity. Nucleic Acids Res. 41, 22–28. doi: 10.1093/nar/gkt389 PMC3692103 23677614

[B59] Langmead B. Salzberg S. L. (2012). Fast gapped-read alignment with Bowtie 2. Nat. Methods 9, 357–359. doi: 10.1038/nmeth.1923 22388286 PMC3322381

[B60] Levy Karin E. Mirdita M. Söding J. (2020). MetaEuk-sensitive, high-throughput gene discovery, and annotation for large-scale eukaryotic metagenomics. Microbiome 8. doi: 10.1186/s40168-020-00808-x PMC7126354 32245390

[B61] Leya T. (2004). Field studies and genetic investigations of the cryophilic snow algae of northwest Spitzbergen (Berlin: Humboldt-Universitaet zu Berlin).

[B62] Li H. Handsaker B. Wysoker A. Fennell T. Ruan J. Homer N.. (2009). The sequence alignment/map format and SAMtools. Bioinformatics 25, 2078–2079. doi: 10.1093/bioinformatics/btp352 19505943 PMC2723002

[B63] Lie A. A. Y. Liu Z. Terrado R. Tatters A. O. Heidelberg K. B. Caron D. A. (2018). A tale of two mixotrophic chrysophytes: Insights into the metabolisms of two Ochromonas species (Chrysophyceae) through a comparison of gene expression. PloS One 13. doi: 10.1371/journal.pone.0192439 PMC5811012 29438384

[B64] Ling H. U. Seppelt R. D. (1990). Snow algae of the Windmill Islands, continental Antarctica. Mesotaenium berggrenii (Zygnematales, Chlorophyta) the alga of grey snow. Antarct. Sci. 2, 143–148. doi: 10.1017/S0954102090000189

[B65] Love M. I. Huber W. Anders S. (2014). Moderated estimation of fold change and dispersion for RNA-seq data with DESeq2. Genome Biol. 15. doi: 10.1186/s13059-014-0550-8 PMC4302049 25516281

[B66] Lyon B. R. Mock T. (2014). Polar microalgae: New approaches towards understanding adaptations to an extreme and changing environment. Biol. (Basel). 3, 56–80. doi: 10.3390/biology3010056 PMC4009763 24833335

[B67] Margesin R. Collins T. (2019). Microbial ecology of the cryosphere (glacial and permafrost habitats): current knowledge. Appl. Microbiol. Biotechnol. 103, 2537–2549. doi: 10.1007/s00253-019-09631-3 30719551 PMC6443599

[B68] McCutcheon J. Lutz S. Williamson C. Cook J. M. Tedstone A. J. Vanderstraeten A.. (2021). Mineral phosphorus drives glacier algal blooms on the Greenland Ice Sheet. Nat. Commun. 12, 570. doi: 10.1038/s41467-020-20627-w 33495440 PMC7835244

[B69] McKie-Krisberg Z. M. Sanders R. W. Gast R. J. (2018). Evaluation of mixotrophy-associated gene expression in two species of polar marine algae. Front. Mar. Sci. 5. 10.3389/fmars.2018.00273

[B70] Mitra A. Flynn K. J. Burkholder J. M. Berge T. Calbet A. Raven J. A.. (2014). The role of mixotrophic protists in the biological carbon pump. Biogeosciences 11, 995–1005. doi: 10.5194/bg-11-995-2014

[B71] Musilova M. Tranter M. Wadham J. Telling J. Tedstone A. Anesio A. M (2017). Microbially driven export of labile organic carbon from the Greenland ice sheet. Nat. Geosci. 10, 360–365. doi: 10.1038/ngeo2920

[B72] Nakanishi H. Seto K. Takeuchi N. Kagami M. (2023). Novel parasitic chytrids infecting snow algae in an alpine snow ecosystem in Japan. Front. Microbiol. 14. doi: 10.3389/fmicb.2023.1201230 PMC10318532 37408638

[B73] Nannipieri P. Giagnoni L. Landi L. Renella G. (2010). “Role of phosphatase enzymes in soil,” in Phosphorus in Action. Eds. Bünemann E. Oberson A. Frossard E. (Springer Berlin Heidelberg, Berlin, Heidelberg), 215–243. doi: 10.1007/978-3-642-15271-9

[B74] Nicholes M. J. Williamson C. J. Tranter M. Holland A. Poniecka E. Yallop M. L.. (2019). Bacterial dynamics in supraglacial habitats of the Greenland ice sheet. Front. Microbiol. 10. doi: 10.3389/fmicb.2019.01366 PMC6616251 31333595

[B75] Oberwinkler F. (2017). Yeasts in pucciniomycotina. Mycol. Prog. 16, 831–856. doi: 10.1007/s11557-017-1327-8

[B76] Parks D. H. Imelfort M. Skennerton C. T. Hugenholtz P. Tyson G. W. (2015). CheckM: Assessing the quality of microbial genomes recovered from isolates, single cells, and metagenomes. Genome Res. 25, 1043–1055. doi: 10.1101/gr.186072.114 25977477 PMC4484387

[B77] Perini L. Gostinčar C. Anesio A. M. Williamson C. Tranter M. Gunde-Cimerman N. (2019). Darkening of the Greenland ice sheet: Fungal abundance and diversity are associated with algal bloom. Front. Microbiol. 10. doi: 10.3389/fmicb.2019.00557 PMC6437116 30949152

[B78] Perini L. Gostinčar C. Likar M. Frisvad J. C. Kostanjšek R. Nicholes M.. (2022). Interactions of fungi and algae from the Greenland ice sheet. Microb. Ecol. 86, 282–296. doi: 10.1007/s00248-022-02033-5 35608637 PMC10293465

[B79] Procházková L. Remias D. Nedbalová L. Raymond J. A. (2024). A DUF3494 ice-binding protein with a root cap domain in a streptophyte glacier ice alga. Front. Plant Sci. 14. doi: 10.3389/fpls.2023.1306511 PMC10796529 38250448

[B80] Procházková L. Řezanka T. Nedbalová L. Remias D. (2021). Unicellular versus filamentous: The glacial alga ancylonema alaskana comb. et stat. nov. and its ecophysiological relatedness to ancylonema nordenskioeldii (zygnematophyceae, streptophyta). Microorganisms 9. doi: 10.3390/microorganisms9051103 PMC8161032 34065466

[B81] Pruesse E. Peplies J. Glöckner F. O. (2012). SINA: Accurate high-throughput multiple sequence alignment of ribosomal RNA genes. Bioinformatics 28, 1823–1829. doi: 10.1093/bioinformatics/bts252 22556368 PMC3389763

[B82] Qiao H. Sun X. R. Wu X. Q. Li G. E. Wang Z. Li D. W. (2019). The phosphate-solubilizing ability of Penicillium guanacastense and its effects on the growth of Pinus massoniana in phosphate-limiting conditions. Biol. Open 8. doi: 10.1242/bio.046797 PMC6899000 31649117

[B83] Quast C. Pruesse E. Yilmaz P. Gerken J. Schweer T. Yarza P.. (2013). The SILVA ribosomal RNA gene database project: Improved data processing and web-based tools. Nucleic Acids Res. 41, 590–596. doi: 10.1093/nar/gks1219 PMC3531112 23193283

[B84] R Core Team. (2021). R: A language and environment for statistical computing. (Vienna, Austria: R Foundation for Statistical Computing). https://www.R-project.org/

[B85] Rassner S. M. E. Cook J. M. Mitchell A. C. Stevens I. T. Irvine-Fynn T. D. L. Hodson A. J.. (2024). The distinctive weathering crust habitat of a High Arctic glacier comprises discrete microbial micro-habitats. Environ. Microbiol. 26. doi: 10.1111/1462-2920.16617 38558266

[B86] Remias D. Schwaiger S. Aigner S. Leya T. Stuppner H. Lütz C. (2012). Characterization of an UV- and VIS-absorbing, purpurogallin-derived secondary pigment new to algae and highly abundant in Mesotaenium berggrenii (Zygnematophyceae, Chlorophyta), an extremophyte living on glaciers. FEMS Microbiol. Ecol. 79, 638–648. doi: 10.1111/j.1574-6941.2011.01245.x 22092588

[B87] Scott D. Hood E. Nassry M. (2010). In-stream uptake and retention of C, N and P in a supraglacial stream. Ann. Glaciol. 51, 80–86. doi: 10.3189/172756411795932065

[B88] Segawa T. Matsuzaki R. Takeuchi N. Akiyoshi A. Navarro F. Sugiyama S.. (2018). Bipolar dispersal of red-snow algae. Nat. Commun. 9. doi: 10.1038/s41467-018-05521-w PMC6079020 30082897

[B89] Stevens I. T. Irvine-Fynn T. D. L. Porter P. R. Cook J. M. Edwards A. Smart M.. (2018). Near-surface hydraulic conductivity of northern hemisphere glaciers. Hydrol. Process. 32, 850–865. doi: 10.1002/hyp.11439

[B90] Stibal M. Schostag M. Cameron K. A. Hansen L. H. Chandler D. M. Wadham J. L.. (2015). Different bulk and active bacterial communities in cryoconite from the margin and interior of the Greenland ice sheet. Environ. Microbiol. Rep. 7, 293–300. doi: 10.1111/1758-2229.12246 25405749

[B91] Sun Y. Liu L. Zeng J. Wu Y. Lin X. (2020). Enhanced cometabolism of benzo(a)anthracene by the lignin monomer vanillate is related to structural and functional responses of the soil microbiome. Soil Biol. Biochem. 149. doi: 10.1016/j.soilbio.2020.107908

[B92] Takeuchi N. Kohshima S. Segawa T. (2003). Effect of cryoconite and snow algal communities on surface albedo on maritime glaciers in south Alaska. Bull. Glaciol. Res. 20, 21–27. Available at: https://www.researchgate.net/publication/267631131.

[B93] Takeuchi N. Tanaka S. Konno Y. Irvine-Fynn T. D. L. Rassner S. M. E. Edwards A. (2019). Variations in phototroph communities on the ablating bare-ice surface of glaciers on brøggerhalvøya, svalbard. Front. Earth Sci. (Lausanne). 7. doi: 10.3389/feart.2019.00004

[B94] Tanabe Y. Shitara T. Kashino Y. Hara Y. Kudoh S. (2011). Utilizing the effective xanthophyll cycle for blooming of ochromonas smithii and O. itoi (chrysophyceae) on the snow surface. PloS One 6. doi: 10.1371/journal.pone.0014690 PMC3044130 21373183

[B95] Tedstone A. J. Cook J. M. Williamson C. J. Hofer S. McCutcheon J. Irvine-Fynn T.. (2020). Algal growth and weathering crust state drive variability in western Greenland Ice Sheet ice albedo. Cryosphere 14, 521–538. doi: 10.5194/tc-14-521-2020

[B96] Varliero G. Lebre P. H. Frey B. Fountain A. G. Anesio A. M. Cowan D. A. (2023). Glacial water: A dynamic microbial medium. Microorganisms 11. doi: 10.3390/microorganisms11051153 PMC10220570 37317127

[B97] Wadham J. L. Hawkings J. R. Tarasov L. Gregoire L. J. Spencer R. G. M. Gutjahr M.. (2019). Ice sheets matter for the global carbon cycle. Nat. Commun. 10, 3567. doi: 10.1038/s41467-019-11394-4 31417076 PMC6695407

[B98] Wadham J. L. Hawkings J. Telling J. Chandler D. Alcock J. O’donnell E.. (2016). Sources, cycling and export of nitrogen on the Greenland Ice Sheet. Biogeosciences 13, 6339–6352. doi: 10.5194/bg-13-6339-2016

[B99] Ward D. M. Bateson M. M. Ferris M. J. Kühl M. Wieland A. Koeppel A.. (2006). Cyanobacterial ecotypes in the microbial mat community of Mushroom Spring (Yellowstone National Park, Wyoming) as species-like units linking microbial community composition, structure and function. Philos. Trans. R. Soc. B.: Biol. Sci. (Royal. Society). 361, 1997–2008. doi: 10.1098/rstb.2006.1919 PMC1764927 17028085

[B100] West P. T. Probst A. J. Grigoriev I. V. Thomas B. C. Banfield J. F. (2018). Genome-reconstruction for eukaryotes from complex natural microbial communities. Genome Res. 28, 569–580. doi: 10.1101/gr.228429.117 29496730 PMC5880246

[B101] Williamson C. J. Anesio A. M. Cook J. Tedstone A. Poniecka E. Holland A.. (2018). Ice algal bloom development on the surface of the Greenland Ice Sheet. FEMS Microbiol. Ecol. 94. 10.1093/femsec/fiy025 PMC6018781 29444265

[B102] Williamson C. J. Cook J. Tedstone A. Yallop M. McCutcheon J. Poniecka E.. (2020). Algal photophysiology drives darkening and melt of the Greenland Ice Sheet. Proc. Natl. Acad. Sci. 117, 5694–5705. doi: 10.1073/pnas.1918412117 32094168 PMC7084142

[B103] Wu X. Rensing C. Han D. Xiao K.-Q. Dai Y. Tang Z.. (2022). Genome-resolved metagenomics reveals distinct phosphorus acquisition strategies between soil microbiomes. mSystems 7. doi: 10.1128/msystems.01107-21 PMC8751388 35014868

[B104] Yallop M. L. Anesio A. M. Perkins R. G. Cook J. Telling J. Fagan D.. (2012). Photophysiology and albedo-changing potential of the ice algal community on the surface of the Greenland ice sheet. ISME. J. 6, 2302–2313. doi: 10.1038/ismej.2012.107 23018772 PMC3504962

[B105] Yoshimura Y. Kohshima S. Ohtani S. (1997). A community of snow algae on a himalayan glacier: change of algal biomass and community structure with altitude. Arctic. Alpine. Res. 29, 126–137. doi: 10.1080/00040851.1997.12003222

[B106] Zawierucha K. Kolicka M. Takeuchi N. Kaczmarek L. (2015). What animals can live in cryoconite holes? A faunal review. J. Zool. 295, 159–169. doi: 10.1111/jzo.12195

[B107] Zheng B. X. Bi Q. F. Hao X. L. Zhou G. W. Yang X. R. (2017). Massilia phosphatilytica sp. nov., a phosphate solubilizing bacteria isolated from a long-term fertilized soil. Int. J. Syst. Evol. Microbiol. 67, 2514–2519. doi: 10.1099/ijsem.0.001916 28853679

