# Supplementary material for: Metagenomic inference of microbial community composition and function in the weathering crust aquifer of a temperate glacier
Source: Front Microbiomes. 2024 Nov 13;3:1488744. doi: 10.3389/frmbi.2024.1488744 (PMC12993576; doi:10.3389/frmbi.2024.1488744)
Supplement: Supplementary file 1 [file DataSheet1.docx]

Metagenomic Inference of Microbial Community Composition and Function in the Weathering Crust Aquifer of a Temperate Glacier

**Quincy Faber^1^, Christina Davis^1†^, Brent Christner^1*^**

^1^University of Florida, Department of Microbiology and Cell Science, Gainesville, FL, USA

^†^Current address: McGill University, Department of Natural Resource Sciences, Sainte-Anne-de-Bellevue, QC, Canada

*** Correspondence:**Brent Christner
xner@ufl.edu

Supplementary Material

# Supplementary Tables

**Table S1.** Sample information.

| Name | Depth (m) | Classification | Year | Amplification | DNA concentration (ng/µl) | Post-amplification DNA concentration (ng/µl) | Library Preparation (ng) | Raw sequence count | Trimmed sequence count |
| --- | --- | --- | --- | --- | --- | --- | --- | --- | --- |
| BH01c_rep1 | 10 | Englacial | 2014 | yes | 0.136 | 0.27 | 2.72 | 387734 | 377251 |
| BH02b_rep1 | 15 | Englacial | 2014 | no | 5.28 | NA | 52.8 | 3012971 | 2919775 |
| BH003_rep1 | 4 | Near-surface | 2014 | no | 4.11 | NA | 41.1 | 2007588 | 1942631 |
| BH04a_rep1 | 4 | Near-surface | 2014 | yes | 0.12 | 0.24 | 2.4 | 106128 | 99560 |
| BH04b_rep1 | 4 | WCA | 2014 | yes | 0.203 | 0.30 | 3.045 | 11080441 | 10683042 |
| Sp001_rep1 | NA | Supraglacial | 2014 | no | 1.69 | NA | 16.9 | 2050128 | 1980999 |
| BH06a_rep1 | 4.4-5.2 | englacial | 2015 | yes | 0.411 | 0.62 | 6.165 | 18692578 | 18029405 |
| BH06b_rep1 | 5.3-5.5 | englacial | 2015 | yes | 0.105 | 0.21 | 2.1 | 91495 | 88409 |
| BH06c_rep1 | 8.3-9.8 | englacial | 2015 | yes | 0.18 | 0.36 | 3.6 | 6671094 | 6388618 |
| BH07a_rep1 | 1.1-4.4 | Near-surface | 2015 | yes | 0.039 | 0.10 | 0.975 | 283309 | 272520 |
| BH07c_rep1 | 11.1-15.4 | Englacial | 2015 | no | 0.059 | NA | 1.475 | 1225355 | 1165459 |
| BH008_rep1 | 5 | Near-surface | 2015 | no | 3.53 | NA | 35.3 | 2842144 | 2731894 |
| BH009_rep1 | 4 | Near-surface | 2015 | no | 1.71 | NA | 17.1 | 822297 | 790100 |
| BH10a_rep1 | 4 | Near-surface | 2015 | no | 0.177 | NA | 3.54 | 939668 | 909932 |
| BH10b_rep1 | 4 | WCA | 2015 | no | 6.18 | NA | 61.8 | 1795746 | 1736103 |
| BH10c_rep1 | 4 | WCA | 2015 | no | 6.21 | NA | 62.1 | 1738331 | 1678240 |
| BH10d_rep1 | 4 | WCA | 2015 | no | 0.284 | NA | 4.26 | 314460 | 300166 |
| BH01c_rep2 | 10 | Englacial | 2014 | no | 0.136 | NA | 2.72 | 1458678 | 1413865 |
| BH02b_rep2 | 15 | Englacial | 2014 | no | 5.28 | NA | 52.8 | 9885926 | 9560646 |
| BH003_rep2 | 4 | Near-surface | 2014 | no | 4.11 | NA | 41.1 | 2943967 | 2844067 |
| BH04a_rep2 | 4 | Near-surface | 2014 | no | 0.12 | NA | 2.4 | 74722 | 60773 |
| BH04b_rep2 | 4 | WCA | 2014 | yes | 0.203 | 0.30 | 3.045 | 6246008 | 6013747 |
| Sp001_rep2 | NA | Supraglacial | 2014 | no | 1.69 | NA | 16.9 | 632898 | 610613 |
| BH06a_rep2 | 4.4-5.2 | Englacial | 2015 | yes | 0.411 | 0.49 | 4.932 | 463888 | 447660 |
| BH06b_rep2 | 5.3-5.5 | Englacial | 2015 | yes | 0.105 | 0.11 | 1.05 | 370068 | 356256 |
| BH06c_rep2 | 8.3-9.8 | Englacial | 2015 | yes | 0.18 | 0.36 | 3.6 | 2888096 | 2771962 |
| BH07a_rep2 | 1.1-4.4 | Near-surface | 2015 | yes | 0.039 | 0.10 | 0.975 | 584598 | 557440 |
| BH008_rep2 | 5 | Near-surface | 2015 | no | 3.53 | NA | 88.25 | 2929075 | 2826636 |
| BH009_rep2 | 4 | Near-surface | 2015 | no | 1.71 | NA | 17.1 | 59789489 | 57634959 |
| BH10a_rep2 | 4 | Near-surface | 2015 | no | 0.177 | NA | 1.77 | 951322 | 919934 |
| BH10b_rep2 | 4 | WCA | 2015 | no | 6.18 | NA | 123.6 | 3525143 | 3413666 |
| BH10c_rep2 | 4 | WCA | 2015 | no | 6.21 | NA | 62.1 | 2637391 | 2533871 |
| BH10d_rep2 | 4 | WCA | 2015 | no | 0.284 | NA | 2.84 | 2574501 | 2492543 |
| BH07c_rep3 | 11.1-15.4 | Englacial | 2015 | no | 1.18 | NA | 17.7 | 4076691 | 3921740 |
| BH10a_rep3 | 4 | Near-surface | 2015 | yes | 60.8 | NA | 608 | 3461210 | 3328103 |
| BH10d_rep3 | 4 | WCA | 2015 | no | 71.6 | NA | 716 | 2983925 | 2872973 |
| BH01c_rep3 | 10 | Englacial | 2014 | no | 1.14 | NA | 11.4 | 6536818 | 6350992 |
| BH04a_rep3 | 4 | Near-surface | 2014 | no | 0.402 | NA | 4.02 | 2703637 | 2593201 |
| BH10a_rep4 | 4 | Near-surface | 2015 | no | 6.22 | NA | 62.2 | 1941170 | 1867663 |

**Table S2.** Metagenomic sequencing information**.**

| Sample | Classification | Year | Number of Contigs | Longest Contig | Shortest Contig | #genes predicted by prodigal | #genes assigned function (bacteria) | #genes assigned function (archaea) | #genes predicted by metaeuk | #genes assigned function (eukaryotes) | #genes assigned function (algae) | #genes assigned function (fungi) |
| --- | --- | --- | --- | --- | --- | --- | --- | --- | --- | --- | --- | --- |
| BH01c_rep1 | Englacial | 2014 | 38424 | 15208 | 78 | 41427 | 13306 | 0 | 35163 | 90 | 21 | 64 |
| BH02b_rep1 | Englacial | 2014 | 159598 | 71704 | 78 | 192355 | 58945 | 5 | 172542 | 1152 | 77 | 966 |
| BH003_rep1 | Near-surface | 2014 | 267661 | 73882 | 78 | 271487 | 20994 | 0 | 140834 | 3541 | 665 | 2408 |
| BH04a_rep1 | Near-surface | 2014 | 4028 | 3740 | 78 | 3639 | 143 | 0 | 1270 | 12 | 2 | 5 |
| BH04b_rep1 | WCA | 2014 | 231010 | 24500 | 78 | 264005 | 8147 | 5 | 104193 | 5947 | 985 | 4707 |
| Sp001_rep1 | Supraglacial | 2014 | 379702 | 47081 | 78 | 405584 | 26174 | 0 | 197828 | 2289 | 625 | 1490 |
| BH06a_rep1 | englacial | 2015 | 81700 | 36269 | 78 | 81043 | 6711 | 0 | 42441 | 268 | 63 | 194 |
| BH06b_rep1 | englacial | 2015 | 14522 | 4410 | 78 | 9343 | 353 | 0 | 4461 | 257 | 5 | 9 |
| BH06c_rep1 | englacial | 2015 | 164183 | 32154 | 77 | 194141 | 14489 | 0 | 110276 | 871 | 251 | 377 |
| BH07a_rep1 | Near-surface | 2015 | 34650 | 10722 | 78 | 34812 | 4193 | 0 | 21530 | 195 | 22 | 138 |
| BH07c_rep1 | Englacial | 2015 | 241101 | 22460 | 78 | 259821 | 36250 | 0 | 183135 | 169 | 90 | 48 |
| BH008_rep1 | Near-surface | 2015 | 133450 | 324173 | 78 | 156047 | 22571 | 0 | 106755 | 780 | 215 | 529 |
| BH009_rep1 | Near-surface | 2015 | 43341 | 17158 | 78 | 45062 | 2670 | 0 | 27028 | 271 | 41 | 223 |
| BH10a_rep1 | Near-surface | 2015 | 25550 | 3624 | 78 | 25078 | 3356 | 0 | 18415 | 35 | 22 | 8 |
| BH10b_rep1 | WCA | 2015 | 227163 | 45221 | 78 | 235830 | 36382 | 6 | 152525 | 1506 | 265 | 1117 |
| BH10c_rep1 | WCA | 2015 | 342708 | 27219 | 78 | 331220 | 26112 | 0 | 167792 | 1742 | 687 | 719 |
| BH10d_rep1 | WCA | 2015 | 11492 | 11468 | 78 | 11660 | 660 | 0 | 6579 | 34 | 29 | 3 |
| BH01c_rep2 | Englacial | 2014 | 173828 | 88930 | 78 | 186766 | 43888 | 5 | 132397 | 682 | 312 | 318 |
| BH02b_rep2 | Englacial | 2014 | 384795 | 94682 | 78 | 419194 | 91899 | 0 | 311721 | 2914 | 418 | 1791 |
| BH003_rep2 | Near-surface | 2014 | 432475 | 73882 | 78 | 462197 | 39076 | 0 | 260320 | 4277 | 724 | 3006 |
| BH04a_rep2 | Near-surface | 2014 | 1133 | 503 | 78 | 1027 | 59 | 0 | 553 | 1 | 1 | 0 |
| BH04b_rep2 | WCA | 2014 | 127222 | 55616 | 78 | 157205 | 5477 | 0 | 70582 | 5413 | 2088 | 4591 |
| Sp001_rep2 | Supraglacial | 2014 | 62019 | 8590 | 78 | 65371 | 4965 | 4 | 40630 | 162 | 32 | 123 |
| BH06a_rep2 | Englacial | 2015 | 82861 | 12998 | 78 | 82779 | 6607 | 0 | 44235 | 456 | 145 | 96 |
| BH06b_rep2 | Englacial | 2015 | 58527 | 21783 | 78 | 37806 | 1334 | 0 | 17799 | 984 | 21 | 28 |
| BH06c_rep2 | Englacial | 2015 | 106433 | 20523 | 77 | 126104 | 9855 | 0 | 72980 | 553 | 231 | 173 |
| BH07a_rep2 | Near-surface | 2015 | 84599 | 17717 | 78 | 90165 | 10809 | 0 | 58518 | 302 | 119 | 134 |
| BH008_rep2 | Near-surface | 2015 | 168082 | 371723 | 78 | 208912 | 34404 | 0 | 160385 | 1005 | 178 | 804 |
| BH009_rep2 | Near-surface | 2015 | 2642350 | 321104 | 78 | 3089508 | 232345 | 20 | 1764325 | 17151 | 4699 | 7299 |
| BH10a_rep2 | Near-surface | 2015 | 25883 | 3624 | 78 | 25540 | 3266 | 0 | 18982 | 44 | 27 | 11 |
| BH10b_rep2 | WCA | 2015 | 435809 | 84803 | 78 | 484575 | 74251 | 0 | 343220 | 2114 | 344 | 1585 |
| BH10c_rep2 | WCA | 2015 | 532323 | 59117 | 78 | 7910 | 480 | 0 | 345765 | 1848 | 758 | 814 |
| BH10d_rep2 | WCA | 2015 | 93408 | 64204 | 78 | 103590 | 4356 | 1 | 49133 | 768 | 192 | 518 |
| BH07c_rep3 | Englacial | 2015 | 538010 | 97100 | 78 | 596199 | 65233 | 0 | 395864 | 813 | 385 | 278 |
| BH10a_rep3 | Near-surface | 2015 | 395381 | 82955 | 78 | 410180 | 14772 | 0 | 151537 | 3051 | 668 | 2107 |
| BH10d_rep3 | WCA | 2015 | 363498 | 39748 | 78 | 369292 | 12869 | 0 | 494383 | 11386 | 430 | 10660 |
| BH01c_rep3 | Englacial | 2014 | 543582 | 90182 | 78 | 611068 | 147268 | 0 | 494383 | 1757 | 415 | 1140 |
| BH04a_rep3 | Near-surface | 2014 | 58623 | 19109 | 78 | 58345 | 1667 | 0 | 17758 | 165 | 27 | 19 |
| BH10a_rep4 | Near-surface | 2015 | 305292 | 27212 | 78 | 10230 | 118696 | 0 | 118696 | 9110 | 352 | 8545 |

**Table S3.** 16S and 18S rRNA gene information**.**

| Sample name | Depth(m) | Classification | Mapped 16S reads | Simpson's Index (16S) | Good's Coverage Index(16S) | Mapped 18S reads | Simpson's Index (18S) | Good's Coverage Index (18S) |
| --- | --- | --- | --- | --- | --- | --- | --- | --- |
| BH01c_rep1 | 10 | Englacial | 410 | 0.991779 | 0.595122 | 37 | 0.96859 | 0.162162 |
| BH02b_rep1 | 15 | Englacial | 1882 | 0.983881 | 0.865569 | 107 | 0.970914 | 0.607477 |
| BH003_rep1 | 4 | Near-surface | 751 | 0.996087 | 0.482024 | 938 | 0.985861 | 0.843284 |
| BH04a_rep1 | 4 | Near-surface | 9 | 0.864198 | 0.222222 | 3 | 0.666667 | 0 |
| BH04b_rep1 | 4 | WCA | 344 | 0.980564 | 0.787791 | 1077 | 0.986733 | 0.89415 |
| Sp001_rep1 | NA | Supraglacial | 433 | 0.996176 | 0.39261 | 496 | 0.989944 | 0.697581 |
| BH06a_rep1 | 4.4-5.2 | Englacial | 84 | 0.977608 | 0.5 | 42 | 0.951247 | 0.52381 |
| BH06b_rep1 | 5.3-5.5 | Englacial | 21 | 0.920635 | 0.285714 | 5 | 0.72 | 0.4 |
| BH06c_rep1 | 8.3-9.8 | Englacial | 291 | 0.988982 | 0.683849 | 98 | 0.805289 | 0.765306 |
| BH07a_rep1 | 1.1-4.4 | Near-surface | 92 | 0.985586 | 0.195652 | 40 | 0.94125 | 0.4 |
| BH07c_rep1 | 11.1-15.4 | Englacial | 224 | 0.993264 | 0.334821 | 24 | 0.947917 | 0.125 |
| BH008_rep1 | 5 | Near-surface | 1378 | 0.979573 | 0.902032 | 123 | 0.976667 | 0.650407 |
| BH009_rep1 | 4 | Near-surface | 138 | 0.988973 | 0.23913 | 88 | 0.97624 | 0.511364 |
| BH10a_rep1 | 4 | Near-surface | 219 | 0.994183 | 0.232877 | 75 | 0.977067 | 0.373333 |
| BH10b_rep1 | 4 | WCA | 1393 | 0.99244 | 0.806892 | 153 | 0.987056 | 0.431373 |
| BH10c_rep1 | 4 | WCA | 456 | 0.996162 | 0.432018 | 291 | 0.989832 | 0.639175 |
| BH10d_rep1 | 4 | WCA | 50 | 0.9792 | 0.04 | 44 | 0.960744 | 0.386364 |
| BH01c_rep2 | 10 | Englacial | 1684 | 0.992305 | 0.792755 | 123 | 0.988565 | 0.308943 |
| BH02b_rep2 | 15 | Englacial | 15289 | 0.98385 | 0.954019 | 1081 | 0.97688 | 0.86864 |
| BH003_rep2 | 4 | Near-surface | 785 | 0.995578 | 0.53758 | 1096 | 0.98399 | 0.864051 |
| BH04a_rep2 | 4 | Near-surface | 4 | 0.75 | 0 | 1 | 0 | 0 |
| BH04b_rep2 | 4 | WCA | 139 | 0.97614 | 0.71223 | 1034 | 0.975154 | 0.909091 |
| Sp001_rep2 | NA | Supraglacial | 109 | 0.987459 | 0.284404 | 85 | 0.970796 | 0.576471 |
| BH06a_rep2 | 4.4-5.2 | Englacial | 131 | 0.986889 | 0.381679 | 46 | 0.964083 | 0.347826 |
| BH06b_rep2 | 5.3-5.5 | Englacial | 31 | 0.957336 | 0.322581 | 13 | 0.899408 | 0.307692 |
| BH06c_rep2 | 8.3-9.8 | Englacial | 203 | 0.984591 | 0.605911 | 41 | 0.961333 | 0.317073 |
| BH07a_rep2 | 1.1-4.4 | Near-surface | 133 | 0.984114 | 0.511278 | 15 | 0.897778 | 0.333333 |
| BH008_rep2 | 5 | Near-surface | 1432 | 0.977508 | 0.89595 | 102 | 0.97501 | 0.676471 |
| BH009_rep2 | 4 | Near-surface | 10090 | 0.997321 | 0.896729 | 9732 | 0.988199 | 0.954377 |
| BH10a_rep2 | 4 | Near-surface | 229 | 0.994603 | 0.183406 | 53 | 0.97686 | 0.226415 |
| BH10b_rep2 | 4 | WCA | 1559 | 0.993691 | 0.773573 | 217 | 0.991612 | 0.43318 |
| BH10c_rep2 | 4 | WCA | 426 | 0.996132 | 0.356808 | 251 | 0.986143 | 0.677291 |
| BH10d_rep2 | 4 | WCA | 50 | 0.9752 | 0.18 | 87 | 0.964196 | 0.689655 |
| BH07c_rep3 | 11.1-15.4 | Englacial | 863 | 0.995804 | 0.691773 | 120 | 0.985 | 0.483333 |
| BH10a_rep3 | 4 | Near-surface | 164 | 0.989218 | 0.414634 | 198 | 0.982196 | 0.757576 |
| BH10d_rep3 | 4 | WCA | 106 | 0.98042 | 0.415094 | 210 | 0.986893 | 0.585714 |
| BH01c_rep3 | 10 | Englacial | 4187 | 0.991727 | 0.867208 | 330 | 0.992544 | 0.572727 |
| BH04a_rep3 | 4 | Near-surface | 27 | 0.949246 | 0.259259 | 20 | 0.905 | 0.45 |
| BH10a_rep4 | 4 | Near-surface | 100 | 0.9782 | 0.47 | 128 | 0.984497 | 0.46875 |

**Table S4.** Top 20 ASVs for 16S rRNA genes **(A)** and 18S rRNA genes **(B).**

**A.**

| 16S | domain | phylum | class | order | family | genus |
| --- | --- | --- | --- | --- | --- | --- |
| OTU 1 | Bacteria | Pseudomonadota | Gammaproteobacteria | Pseudomonadales | Pseudomonadaceae | Pseudomonas |
| OTU 2 | Bacteria | Pseudomonadota | Gammaproteobacteria | NA | NA | NA |
| OTU 3 | Bacteria | Pseudomonadota | Gammaproteobacteria | Pseudomonadales | Pseudomonadaceae | Pseudomonas |
| OTU 4 | Bacteria | Pseudomonadota | Gammaproteobacteria | Pseudomonadales | Pseudomonadaceae | Pseudomonas |
| OTU 5 | Bacteria | Pseudomonadota | Gammaproteobacteria | Pseudomonadales | Pseudomonadaceae | Pseudomonas |
| OTU 6 | Bacteria | Pseudomonadota | Gammaproteobacteria | Pseudomonadales | Pseudomonadaceae | Pseudomonas |
| OTU 7 | Bacteria | Pseudomonadota | Gammaproteobacteria | Pseudomonadales | Pseudomonadaceae | Pseudomonas |
| OTU 8 | Bacteria | Pseudomonadota | Gammaproteobacteria | Pseudomonadales | Pseudomonadaceae | Pseudomonas |
| OTU 9 | Bacteria | Pseudomonadota | Gammaproteobacteria | Pseudomonadales | Pseudomonadaceae | NA |
| OTU 10 | Bacteria | Pseudomonadota | Gammaproteobacteria | Pseudomonadales | Pseudomonadaceae | Pseudomonas |
| OTU 11 | Bacteria | Pseudomonadota | Gammaproteobacteria | Pseudomonadales | Pseudomonadaceae | Pseudomonas |
| OTU 12 | Bacteria | Pseudomonadota | Gammaproteobacteria | Pseudomonadales | Pseudomonadaceae | Pseudomonas |
| OTU 13 | Bacteria | Pseudomonadota | Gammaproteobacteria | Pseudomonadales | Pseudomonadaceae | Pseudomonas |
| OTU 14 | Bacteria | Pseudomonadota | Gammaproteobacteria | Pseudomonadales | Pseudomonadaceae | Pseudomonas |
| OTU 15 | Bacteria | Pseudomonadota | Gammaproteobacteria | Pseudomonadales | Pseudomonadaceae | Pseudomonas |
| OTU 16 | Bacteria | Pseudomonadota | Gammaproteobacteria | Pseudomonadales | Pseudomonadaceae | Pseudomonas |
| OTU 17 | Bacteria | Pseudomonadota | Betaproteobacteria | Burkholderiales | Oxalobacteraceae | NA |
| OTU 18 | Bacteria | Pseudomonadota | Gammaproteobacteria | Pseudomonadales | Pseudomonadaceae | Pseudomonas |
| OTU 19 | Bacteria | Pseudomonadota | Gammaproteobacteria | Pseudomonadales | Pseudomonadaceae | Pseudomonas |
| OTU 20 | Bacteria | Pseudomonadota | Gammaproteobacteria | Pseudomonadales | Pseudomonadaceae | Pseudomonas |

**B.**

|  | domain | phylum | class | order | family | genus |
| --- | --- | --- | --- | --- | --- | --- |
| OTU 1 | Eukaryota | Basidiomycota | Microbotryomycetes | NA | NA | NA |
| OTU 2 | Eukaryota | Basidiomycota | Microbotryomycetes | NA | NA | NA |
| OTU 3 | Eukaryota | Ascomycota | Dothideomycetes | NA | NA | NA |
| OTU 4 | Eukaryota | NA | NA | NA | NA | NA |
| OTU 5 | Eukaryota | Basidiomycota | NA | NA | NA | NA |
| OTU 6 | Eukaryota | NA | NA | NA | NA | NA |
| OTU 7 | Eukaryota | Basidiomycota | Microbotryomycetes | NA | NA | NA |
| OTU 8 | Eukaryota | Basidiomycota | Microbotryomycetes | NA | NA | NA |
| OTU 9 | Eukaryota | Basidiomycota | NA | NA | NA | NA |
| OTU 10 | Eukaryota | Charophyta | Zygnematophyceae | Zygnematophyceae | Zygnematophyceae | Zygnematophyceae |
| OTU 11 | Eukaryota | Basidiomycota | Microbotryomycetes | Incertae_Sedis | Chrysozymaceae | NA |
| OTU 12 | Eukaryota | Basidiomycota | NA | NA | NA | NA |
| OTU 13 | Eukaryota | Basidiomycota | Microbotryomycetes | NA | NA | NA |
| OTU 14 | Eukaryota | NA | NA | NA | NA | NA |
| OTU 15 | Eukaryota | Basidiomycota | Microbotryomycetes | NA | NA | NA |
| OTU 16 | Eukaryota | Charophyta | Zygnematophyceae | Zygnematophyceae | Zygnematophyceae | Zygnematophyceae |
| OTU 17 | Eukaryota | Ochrophyta | Chrysophyceae | NA | NA | NA |
| OTU 18 | Eukaryota | Basidiomycota | Microbotryomycetes | NA | NA | NA |
| OTU 19 | Eukaryota | NA | NA | NA | NA | NA |
| OTU 20 | Eukaryota | Charophyta | Embryophyta | Magnoliophyta | Magnoliophyta | Magnoliophyta |

# Supplementary Figures


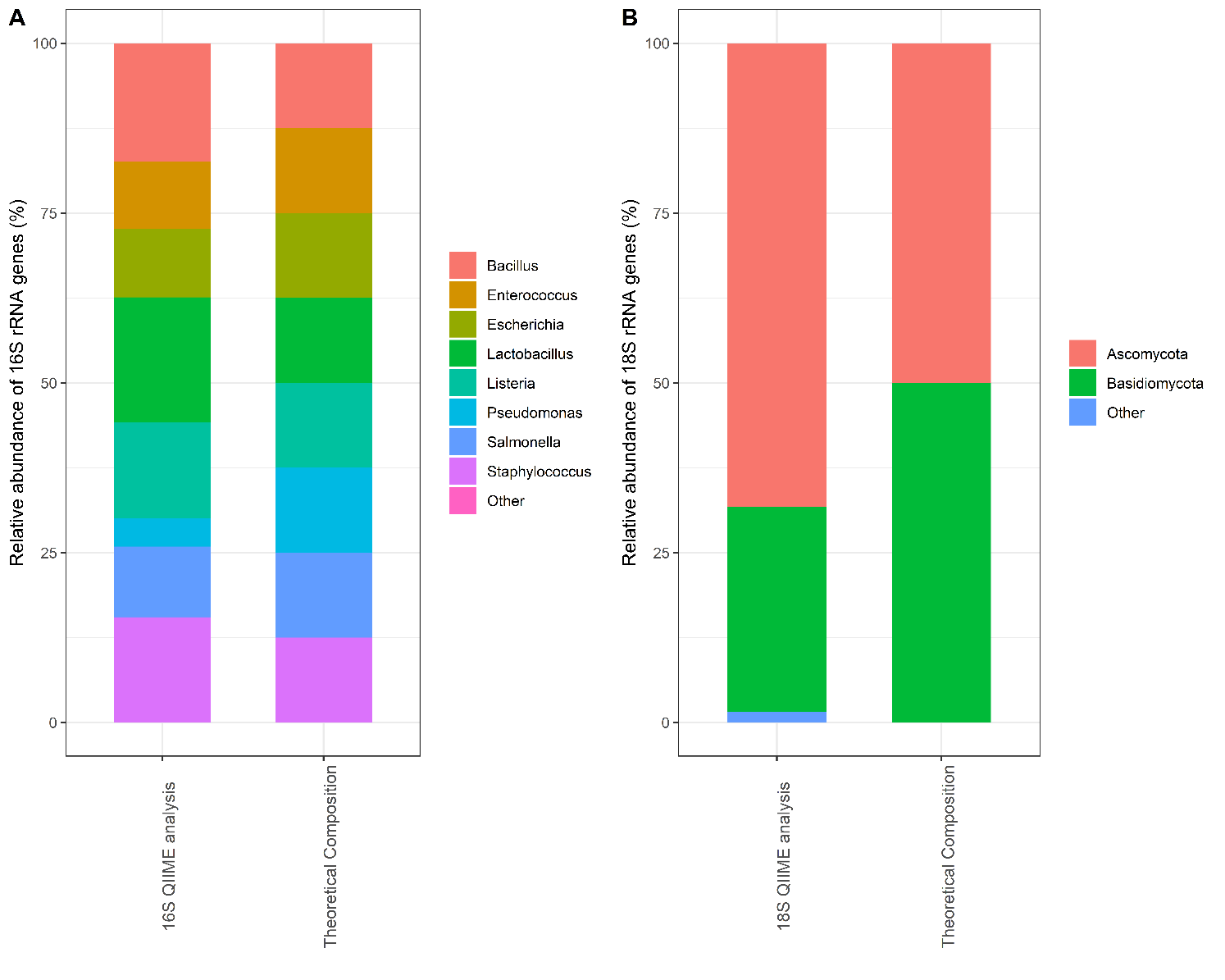


**Figure S1.** Relative % abundance of 16S rRNA genes **(A)** and 18S rRNA genes **(B)** for a mock community versus theoretical composition. The mock community used was ZymoBIOMICS™ Microbial Community Standard Catalog No. D6300.


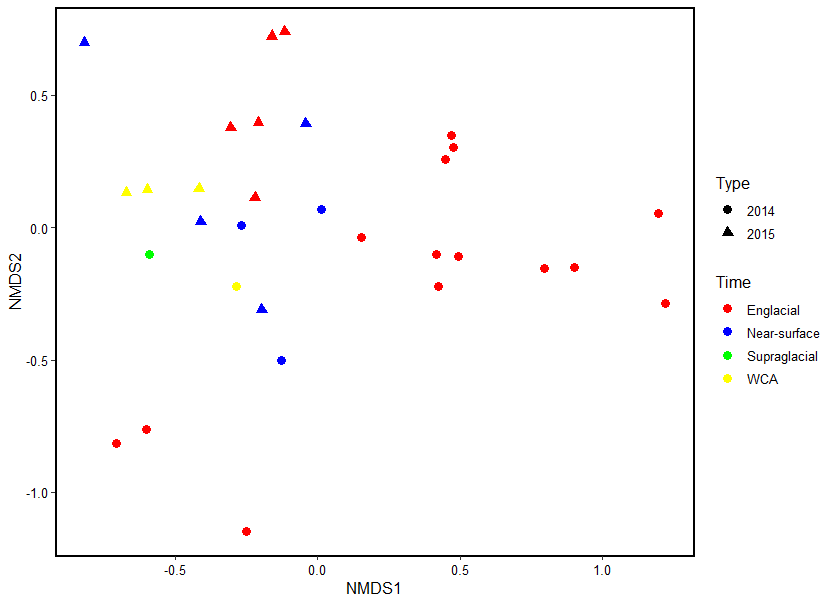


**Figure S2.** NMDS of Bray-Curtis distance among samples collected during 2014 and 2015 from the supraglacial stream, WCA, and ice from the near-surface and englacial zone based on 16S rRNA gene amplicon sequencing (stress= 0.1845; Christner et al., 2018). The plot was generated using ggplot2 in R.


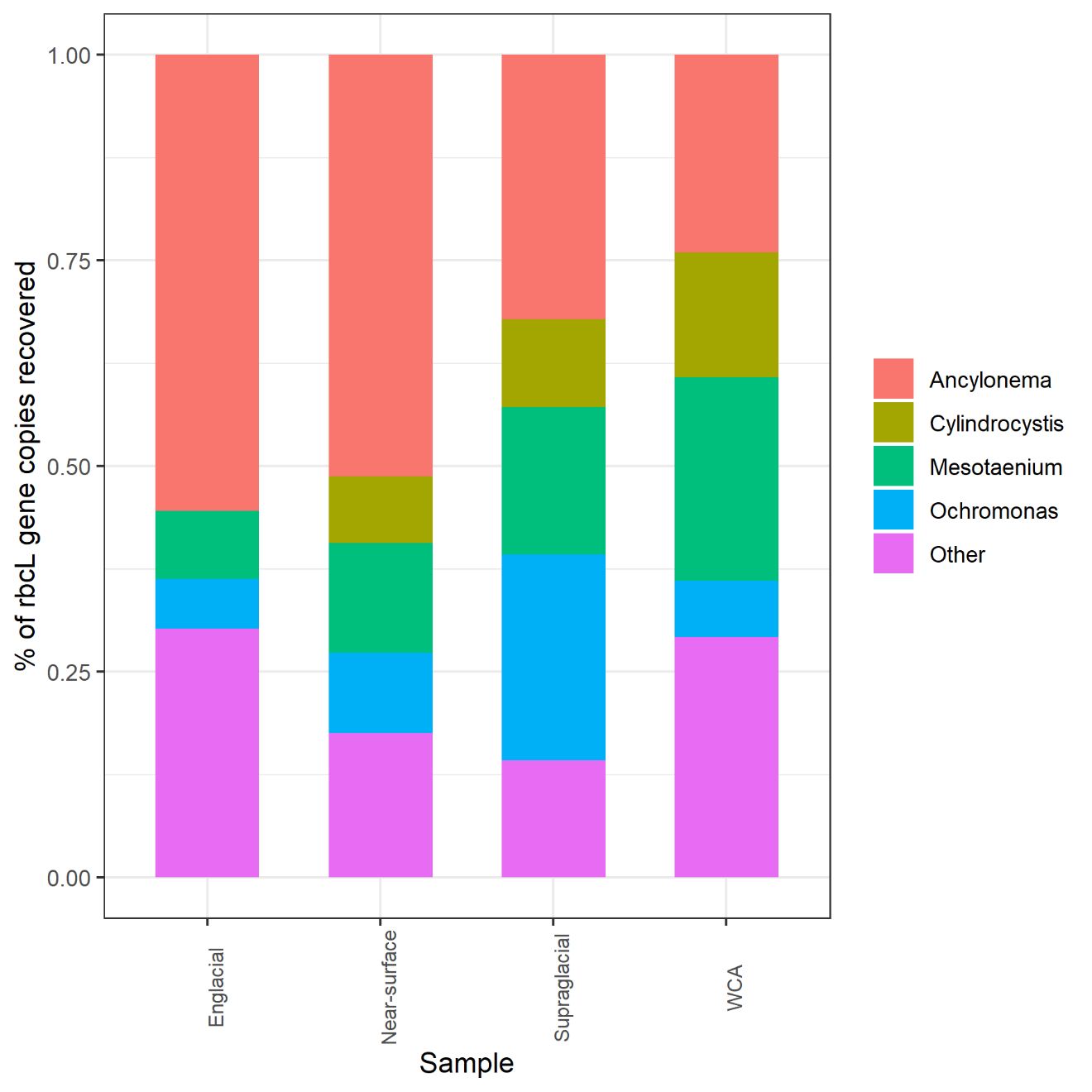


**Figure S3.** Relative abundances of chloroplast-containing organisms based on *rbcL* genes.


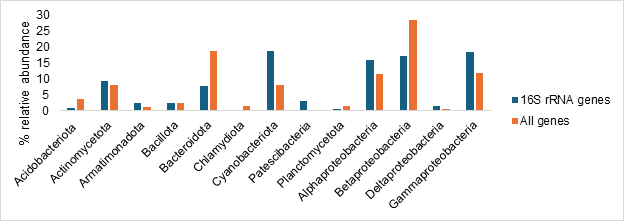

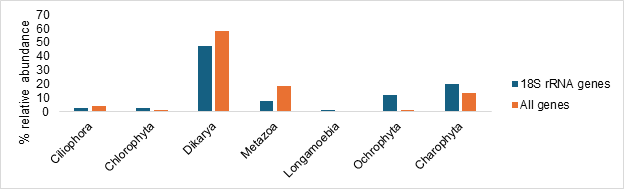


**Figure S4. (A)** Relative abundances of prokaryotic taxa from 16S rRNA genes and assembled functional genes. **(B)** Relative abundances of eukaryotic taxa from 18S rRNA genes and assembled functional genes.


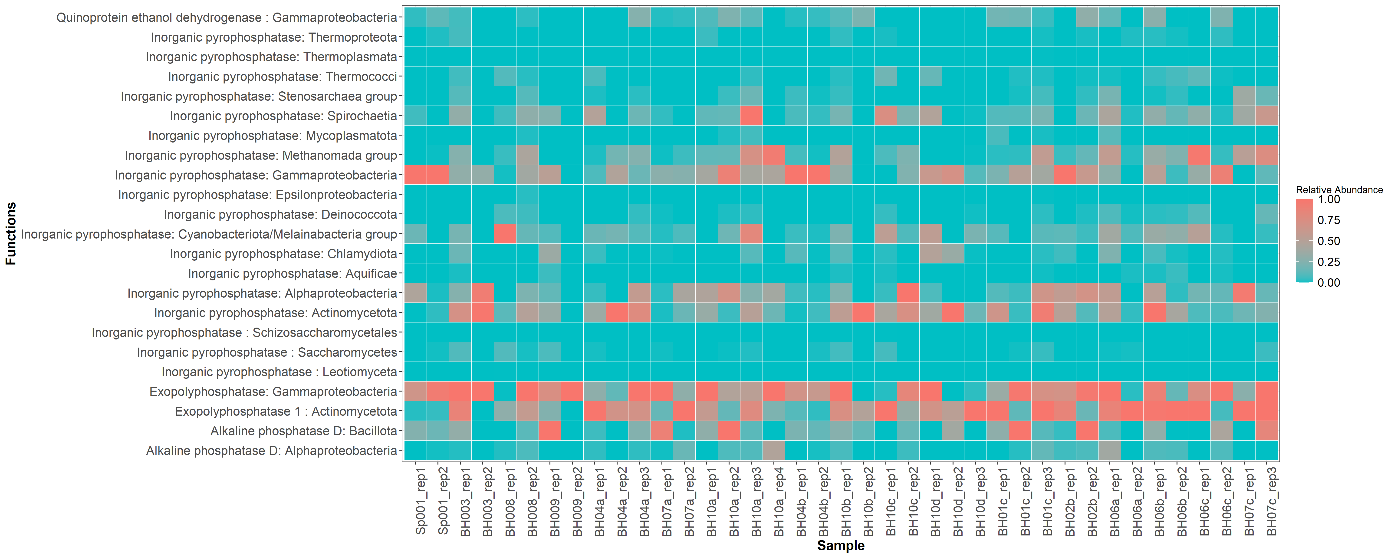
 **Figure S5.** Phosphorus cycling genes found across samples.
